# Hierarchical discovery of large-scale and focal copy number alterations in low-coverage cancer genomes

**DOI:** 10.1186/s12859-020-3480-3

**Published:** 2020-04-16

**Authors:** Ahmed Ibrahim Samir Khalil, Costerwell Khyriem, Anupam Chattopadhyay, Amartya Sanyal

**Affiliations:** 1 School of Computer Science and Engineering, Nanyang Technological University, 50 Nanyang Avenue, Singapore, 639798 Singapore; 2 School of Biological Sciences, Nanyang Technological University, 60 Nanyang Drive, Singapore, 637551 Singapore

**Keywords:** Cancer, DNA copy number alteration, Focal amplification and deletion, Segmental aneuploidy, Genome sequence analysis, Read depth, Copy number detection tool, Simulated copy number profile

## Abstract

**Background:**

Detection of DNA copy number alterations (CNAs) is critical to understand genetic diversity, genome evolution and pathological conditions such as cancer. Cancer genomes are plagued with widespread multi-level structural aberrations of chromosomes that pose challenges to discover CNAs of different length scales, and distinct biological origins and functions. Although several computational tools are available to identify CNAs using read depth (RD) signal, they fail to distinguish between large-scale and focal alterations due to inaccurate modeling of the RD signal of cancer genomes. Additionally, RD signal is affected by overdispersion-driven biases at low coverage, which significantly inflate false detection of CNA regions.

**Results:**

We have developed CNAtra framework to hierarchically discover and classify ‘large-scale’ and ‘focal’ copy number gain/loss from a single whole-genome sequencing (WGS) sample. CNAtra first utilizes a multimodal-based distribution to estimate the copy number (CN) reference from the complex RD profile of the cancer genome. We implemented Savitzky-Golay smoothing filter and Modified Varri segmentation to capture the change points of the RD signal. We then developed a CN state-driven merging algorithm to identify the large segments with distinct copy numbers. Next, we identified focal alterations in each large segment using coverage-based thresholding to mitigate the adverse effects of signal variations. Using cancer cell lines and patient datasets, we confirmed CNAtra’s ability to detect and distinguish the segmental aneuploidies and focal alterations. We used realistic simulated data for benchmarking the performance of CNAtra against other single-sample detection tools, where we artificially introduced CNAs in the original cancer profiles. We found that CNAtra is superior in terms of precision, recall and f-measure. CNAtra shows the highest sensitivity of 93 and 97% for detecting large-scale and focal alterations respectively. Visual inspection of CNAs revealed that CNAtra is the most robust detection tool for low-coverage cancer data.

**Conclusions:**

CNAtra is a single-sample CNA detection tool that provides an analytical and visualization framework for CNA profiling without relying on any reference control. It can detect chromosome-level segmental aneuploidies and high-confidence focal alterations, even from low-coverage data. CNAtra is an open-source software implemented in MATLAB^®^. It is freely available at https://github.com/AISKhalil/CNAtra.

## Background

DNA copy number alteration (also commonly referred to as copy number variation or CNV) is a generic term broadly used to define genetic variations that lead to the changes in the number of copies of genomic regions. CNA events are gain or loss of DNA regions compared to the reference sample(s) or assembly that are 1 kb or larger in size [1–4]. In cancer, the overwhelming extent of CNA size distribution resulted in their further classification into *microscopic level* ‘large-scale’ or *submicroscopic level* ‘focal’ chromosomal aberrations [3–5]. First, the large-scale copy number variations (LCVs) concern chromosomal abnormalities at Mb scale, such as segmental aneuploidy, that can be cytogenetically detected [4]. Sometimes these LCVs represent polymorphic variations among individuals in a population [6]. Second, the focal alterations (FAs) can range between kb to a few Mb in size containing a small number of genes, believed to harbor important oncotargets [5, 7]. Both LCVs and FAs are pervasive in cancer cell lines, which serve as important pre-clinical models for cancer research, drug screening and discovery [8]. Naturally, accurate detection of both these alteration phenomena is crucial for gaining insights on their origin and biological context. Because of their variable size distribution, the current copy number detection tools generally target a specific range of CNA size [9]. Therefore, detection methodology needs to be tuned to identify the complete spectrum of CNAs (large-scale and focal events) and should include procedures to distinguish them.

Numerous next-generation sequencing (NGS)-based computational tools have been developed for detection of copy number changes by adopting different strategies such as paired-end mapping, split-read, read depth, de novo assembly or combinatorial approaches [9–11]. Among them, the most common and widely-used strategy utilizes depth of coverage from WGS data to identify ‘absolute’ copy number by modelling the RD signal either from an individual sample (single sample), or to discover ‘relative’ copy number by taking advantage of matched normal sample (paired case-control sample) or using samples from multiple subjects/individuals from a healthy population [10]. These RD-based approaches have been successfully used depending on the study design and data availability [9]. Matched control from tumor-adjacent normal tissue is generally difficult to procure. Hence, majority of tumor datasets do not have corresponding tumor-adjacent normal sample in genomic databases, such as The Cancer Genome Atlas (TCGA) [12]. As an alternative, whole blood is commonly used as matched control for solid tumors to control for genetic background, and sometimes non-invasively collected samples (e.g. saliva or buccal samples) are also employed. However, the source of ‘normal’ samples greatly impacts the quality of genome analysis such as copy number detection outcome [13, 14]. Additionally, most of the cancer cell lines do not have the corresponding normal counterpart derived from the same individual. Therefore, single-sample computational tools, which do not rely on matched controls, are applicable for both cancer cell lines as well as patient tumor samples.

Several tools have been presented over the years that utilize a single sequencing sample for copy number detection [15–22]. They are built on different assumptions of the underlying probabilistic distribution and percentage of chromosomal abnormalities. However, the extent of copy number changes is widespread in cancer genomes as they are plagued with large-scale segmental aneuploidies which may lead to inaccurate estimation of the CN reference. Moreover, disregarding the distinction of large-scale and focal events, these single-sample tools suffer from oversegmentation of LCVs and erroneous calling of FAs. Additionally, in low-coverage data, detection of copy number change is adversely affected by overdispersion and short-term variations such as wave artifacts [23–25]. In such scenarios, statistics-based segmentation [15, 20, 26] and CNA calling lead to either false segmentation or missing the FAs. Despite these limitations, CNAs detected using low-coverage clinical samples eclipsed the performance of array-based detection methods [27] suggesting the importance of WGS samples even if available at shallow coverage.

Therefore, in order to address these challenges, we developed CNAtra [**C**opy **N**umber **A**lteration (detection) **t**hrough **r**ead depth **a**nalysis], a MATLAB-based hierarchical computational framework for the sensitive and robust detection of LCVs and FAs. CNAtra is built on the fundamental assumption that most genomic regions of any cell are centralized toward copy number states of positive integer values. CNAtra empirically models the RD signal based on a multimodal distribution and estimate the CN reference. This approach allows us to define the accurate ‘interval’ of CN states which aids in identifying segmental aneuploidies (and FAs within them) in a robust manner largely unaffected by coverage, percentage of karyotype abnormality and wave artifacts. For this, we first applied a robust signal-processing technique of univariate time series to identify significant change points of the RD signal. These change points are used for assembling large segments based on their CN states. These assembled segments are termed as iso-copy number blocks (IBs) and they are used for identifying the candidate FAs within them. In addition, for handling the overdispersion problem of low-coverage data, we incorporated coverage-based thresholding parameters beside the conventional statistical test to identify the significant FAs. CNAtra also provides an interactive platform to visualize and manually inspect the complete (genome-wide) copy number profile and accessory information for further validation, interpretation and downstream application of CNA calls. We successfully verified CNAtra results using experimentally-validated segmental aneuploidies and focal amplifications/deletions across several cancer cell lines. We also applied CNAtra to patient tumor samples and showed that CNAtra successfully detected the copy number changes from a single WGS sample without the requirement of matched control sample. We then benchmarked the performance of CNAtra against five single-sample CN detection tools using realistic simulated data by randomly introducing copy number events in the original cancer genome. The evaluation showed the ability of CNAtra to resolve complex CNA profiles into LCVs and FAs with the highest f-measure. Manual review and visualization also verified the advantage of CNAtra over other single-sample tools.

## Results

### Cancer genomes harbor LCV and FA with distinct biological origins

**Fig. 1:**
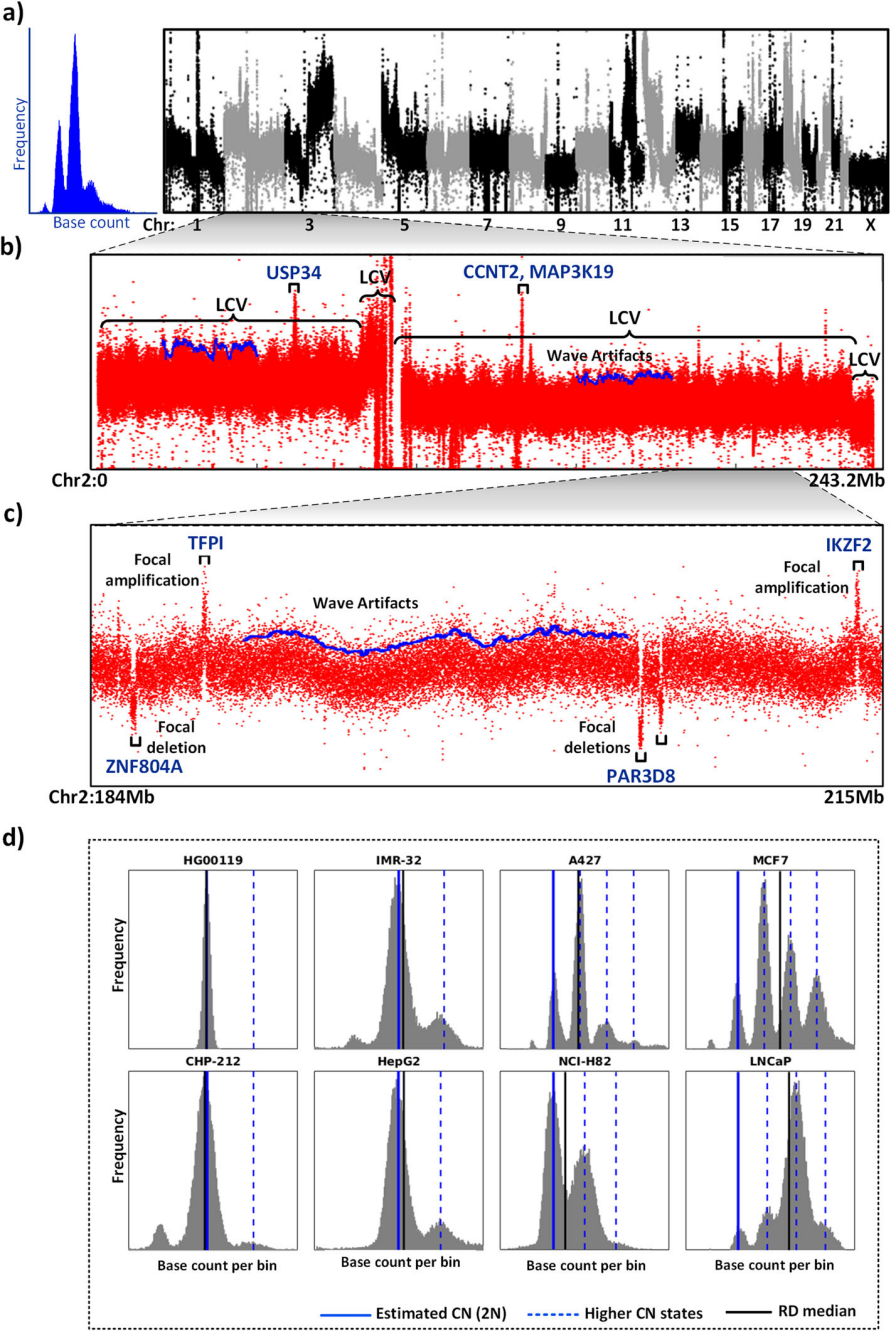
Characterization of RD signal in cancer genome. **a** The RD frequency distribution (left) and genome-wide coverage plot of all chromosomes (right) of A427 cell line showing the presence of widespread LCVs. **b** Coverage plot of chromosome (Chr) 2 showing LCVs of different CN states. **c** Zoomed in view of Chr 2 (184–215 Mb) shows the presence of FAs (both amplifications and deletions) inside the LCV. The blue curved lines in **(b)** and **(c)** denote the upper envelope of RD signal showing the regions affected by wave artifacts. Few loci of focal amplifications and deletions are indicated. **d** RD frequency distributions of a normal genome (HG00119) and different cancer cell lines showing the estimation of the CN reference (2N) by CNAtra (solid blue line). The black line denotes the global RD median, while the dotted blue lines indicate the higher CN states (3N, 4N, 5N) based on CNAtra CN reference

Cancer cells are afflicted with widespread numerical and structural variations of chromosomes which positively correlate with tumor aggressiveness [28, 29]. Cancer cells contain both LCVs and FAs having different mechanisms of origin and functional roles. LCVs are microscopically-visible whole chromosome or large genomic ‘blocks’ (Mb scale) [4] with distinct CN states which results from chromosomal instability leading to acquisition of complex genetic makeup by the cancer cells [30]. In contrast, the focal amplifications and deletions emerge as a consequence of adaptive selection events that facilitate selective growth advantage and evolution of malignant cells during tumor/cancer development and drug resistance [3, 30]. Focal amplifications generally have high-level gains of oncogenes or anti-apoptotic genes, while focal deletions usually involve tumor suppressor or pro-apoptotic genes [31, 32]. Therefore, identification and characterization of these two phenomena can provide vital clues to identify the genomic regions and driver genes involved in carcinogenesis and their roles in cancer evolution. An illustrative example using a coverage plot from WGS data of A427 lung cancer cell line is provided in Fig. 1. LCVs are pervasive in A427 genome which results in the multimodal frequency distribution of RD signal (Fig. 1a). A closer look at chromosome 2 (Fig. 1b) shows that some focal events are interspersed within LCVs creating a complex relationship between them. For example, focal amplifications containing *USP34* and *CCNT2* genes are part of different LCVs in 2p and 2q regions, respectively (Fig. 1b). Therefore, there can be a complex scenario where a genomic region with hemizygous segmental deletion (LCV) may contain a focally-amplified region. In the coverage plot, these LCVs appear as ‘large’ segments, and they are strongly affected by ‘wave artifacts’ (indicated as a blue curve in Fig. 1b,c). Wave artifacts are systematic biases due to deviation from equimolar coverage signal [33]. On the other hand, the focal amplifications and deletions appeared as ‘sharp’ peaks and troughs respectively (Fig. 1b,c). Moreover, RD signal is also prone to inherent biases associated with NGS owing to genome GC content, low-mappability regions and coverage-influenced signal overdispersion. All these biases ultimately complicate the detection of segmental aneuploidies and FAs. Taken together, it can be concluded that cancer genomes have multi-level CNAs and their RD signals are inherently complex as evidenced by their nature and distribution as well as their association with systematic biases. Therefore, copy number detection in cancer genomes necessitates the biological understanding of this underlying complexity and based on which a step-by-step approach needs to be implemented to delineate the multi-level aberrations one at a time. None of the currently-available tools have adequately addressed these multi-level issues in toto.

### Accurate estimation of CN reference is essential for CNA calling in cancer genomes

**Fig. 2:**
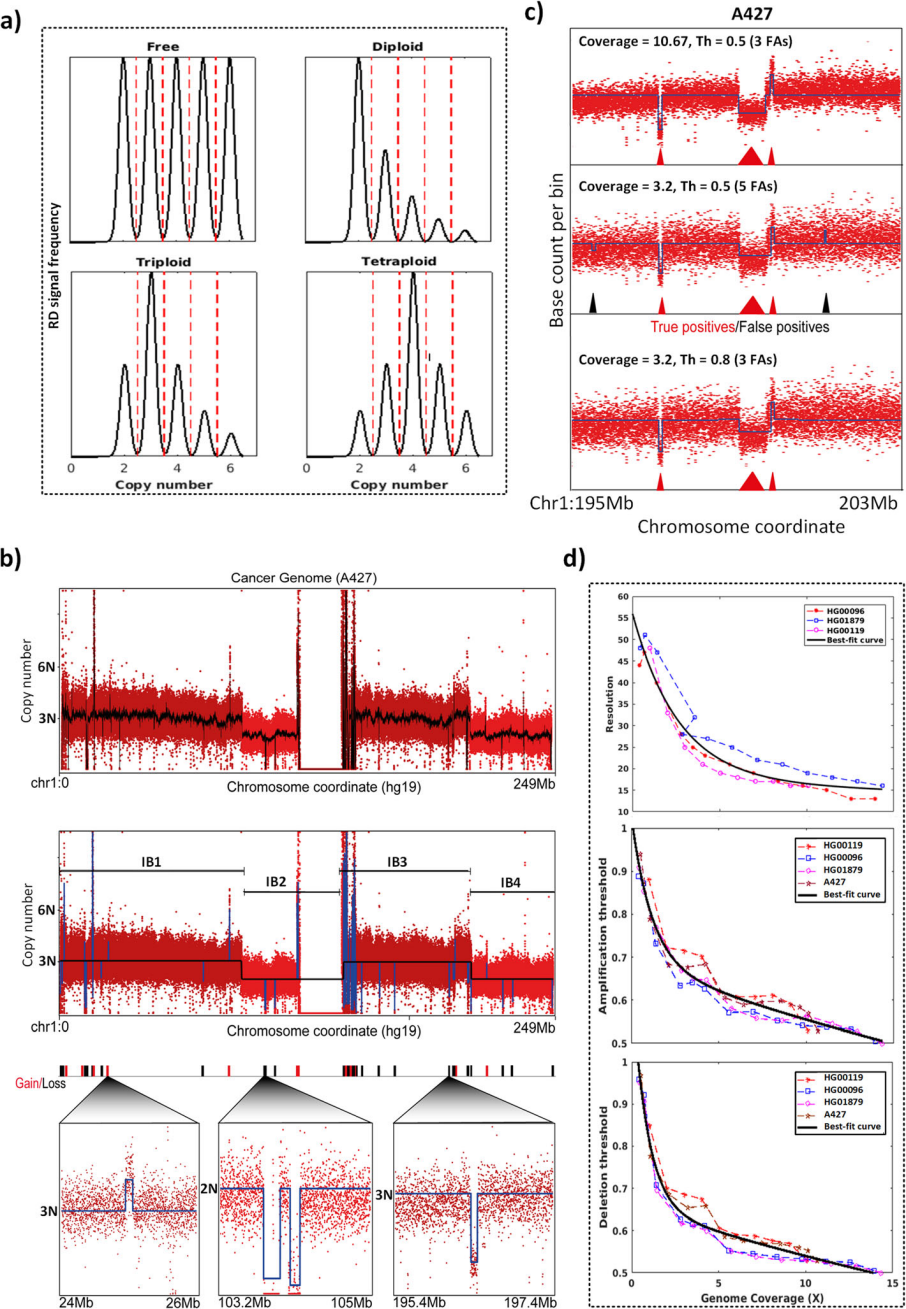
CNAtra solution for multi-level CNA detection in low-coverage data. **a** Schematic representation of multimodal distribution under different ploidy assumptions. Dotted red lines denote the boundaries of CN states (CN intervals). **b** A hierarchical computational framework for detecting both LCVs and FAs. The CNAtra approach includes smoothing of RD signal using Savitzky-Golay filter (black line) (top panel), followed by detection of IBs with distinct CN states (middle panel) and then identification of FAs inside each IB (bottom panel). **c** Effects of tuning the amplification/deletion thresholds (Th) on the detection of false positive FAs in a region in Chr 1 (195–203 Mb) using subsampled data of A427 cell line. The red and black triangles represent the true positives and false positives respectively. **d** Calibration of CNAtra parameters such as resolution (top panel), amplification threshold (middle panel) and deletion threshold (bottom panel) using high-coverage WGS datasets. The solid black line represents the fitted regression line using negative exponential model

In addition to the presence of LCVs, chromosome segregation errors lead to elevated ploidy (at genome or chromosome levels) and karyotype alterations in cancer cells [34, 35]. Human cancer genomes frequently have hyperdiploid, near-triploid or higher ploidy levels [36–38]. All these anomalies manifest as multimodal distribution of the RD signal. We analyzed several cancer cell lines of different ploidy and complexity using publicly available data (Supplementary Table 1). Genomes of cancer cell lines exhibited a complex *multimodal* distribution as opposed to normal genomes (1000 Genomes Project) which follow a *unimodal* distribution (Fig. 1d; Supplementary Figure S1). We also found that medians of RD signals across chromosomes are highly inconsistent for cancer cell lines (Supplementary Figure S1**)**. Most single-sample CN detection tools assume unimodal distribution of the RD signal and use the global median as CN reference (2N). In contrast, CNAtra utilized a multimodal distribution as a summation of normal distributions of different probability centralized at CN states under a given ploidy assumption (Fig. 2a). This allows accurate estimation of the CN reference (2N). As shown in Fig. 1d, our estimated CN reference (2N; solid blue line) and other CN states (dashed blue lines) are coinciding with peaks of the RD signal. However, the global median (black line) deviates from the ‘actual’ CN reference (solid blue line) by 2.5–87% (Supplementary Table 2) depending on the percentage of LCVs in the corresponding cancer cell line, as opposed to 0.34–0.56% for normal genomes.

Nevertheless, karyotype or whole-genome ploidy information is not always available for cancer cell lines. In that case, our ‘free model’ can still predict the CN reference with a maximum error of 0.44% compared to the CN reference computed by other models (Supplementary Table 2). Therefore, our multimodal approach can be effectively applied to cancer cell lines for which karyotype information is largely unknown. Defining the CN reference forms the basis to discover the segmental aneuploidies and to estimate the CN states accurately.

### Hierarchical framework enables CNAtra to identify large-scale and focal alterations

We have taken a pragmatic approach for solving the two major problems associated with cancer genomes- 1) presence of LCVs and 2) systematic biases such as overdispersion and wave artifacts which are pronounced in low-coverage data. We used Savitzky–Golay filter (a weighted moving average filter that smooths out short-term variations and preserves inherent RD features without shifting effect) to successfully attenuate the wave artifacts and signal variability in order to identify the primary segments by Modified Varri segmentation (Fig. 2b top panel; black line). However, copy number detection methods based solely on segmentation may suffer from false segmentation or oversegmentation. For example, neighboring segments may represent copy number altered regions belonging to the same CN state which has no biological basis (false segmentation). In addition, an LCV region can be falsely ‘oversegmented’ into several regions with the same copy number due to the presence of focal amplifications and deletions within it. This may lead to failure in capturing the entire LCV as a single event. We solved this problem using an assembly algorithm which successfully merges primary segments with the same copy number to define the IB (Fig. 2b middle panel). Each IB represents distinct (unimodal) peak in the multimodal RD signal distribution centered on/near a CN state. An IB with CN state different from CN reference is considered as a segmental aneuploidy or LCV.

After defining the IBs, we found that RD signal from each IB follows the normal distribution using Q-Q plot and Kolmogorov–Smirnov test with a reasonably good approximation (5% significance level) (Supplementary Figure S2). Therefore, we use each IB as a population of RD signal bins for the discovery of *statistically significant* FAs (**Class 1)** using the t-test. However, due to the overdispersion of RD signal in cancer genomes, statistical tests may reject the null hypothesis of long segments with a small mean difference (particularly in the presence of wave artifacts) resulting in many false positives. Therefore, we employed coverage-based thresholding to define *high confidence* FAs (**Class 2**) based on the local CN reference of the parent IB (Fig. 2b bottom panel).

### Coverage-based thresholding enables the detection of high confidence focal alterations in low-coverage data

Dispersion of RD signal is inversely proportional to the depth of coverage. Therefore, CN detection tools usually recommend using large bins (coverage-driven bin-size approach) to avoid false detection of CNAs in low-coverage datasets. However, this approach may result in missing true FAs. As an alternative, we propose to adapt the coverage-driven thresholding approach instead of increasing the bin-size. This allows the identification of high confidence FAs without increasing the number of false positives.

Theoretically, any ‘candidate’ FA with amplitude-shift > 0.5N (threshold) from the copy number of its parent IB can be identified as a significant FA since it belongs to another CN state (Supplementary Figure S3). However, low-coverage data suffer from higher RD signal variability resulting in increased false positives with the same threshold (Fig. 2c). Therefore, we define the coverage-based parameters (resolution, amplification and deletion thresholds) to overcome the problem of overdispersion in low-coverage data (Fig. 2d). We utilized negative exponential regression for modeling the relationship between sequencing coverage and the coverage-based parameters using WGS datasets. Our thresholding parameters enable a user to strike a balance between false positives and false negatives. For example, using the same 0.5N as the amplification and deletion thresholds, the subsampling of A427 data to 3.2x coverage yields more false positives compared to the original 10.67x coverage (Fig. 2c; top and middle panel). Increasing these thresholds gets rid of these false positives (Fig. 2c bottom panel). Therefore, the advantage of coverage-based tuning of thresholding parameters makes the CNAtra results more robust at different data coverage.

### CNAtra detects experimentally-validated large-scale and focal alterations across cancer cell lines

**Fig. 3:**
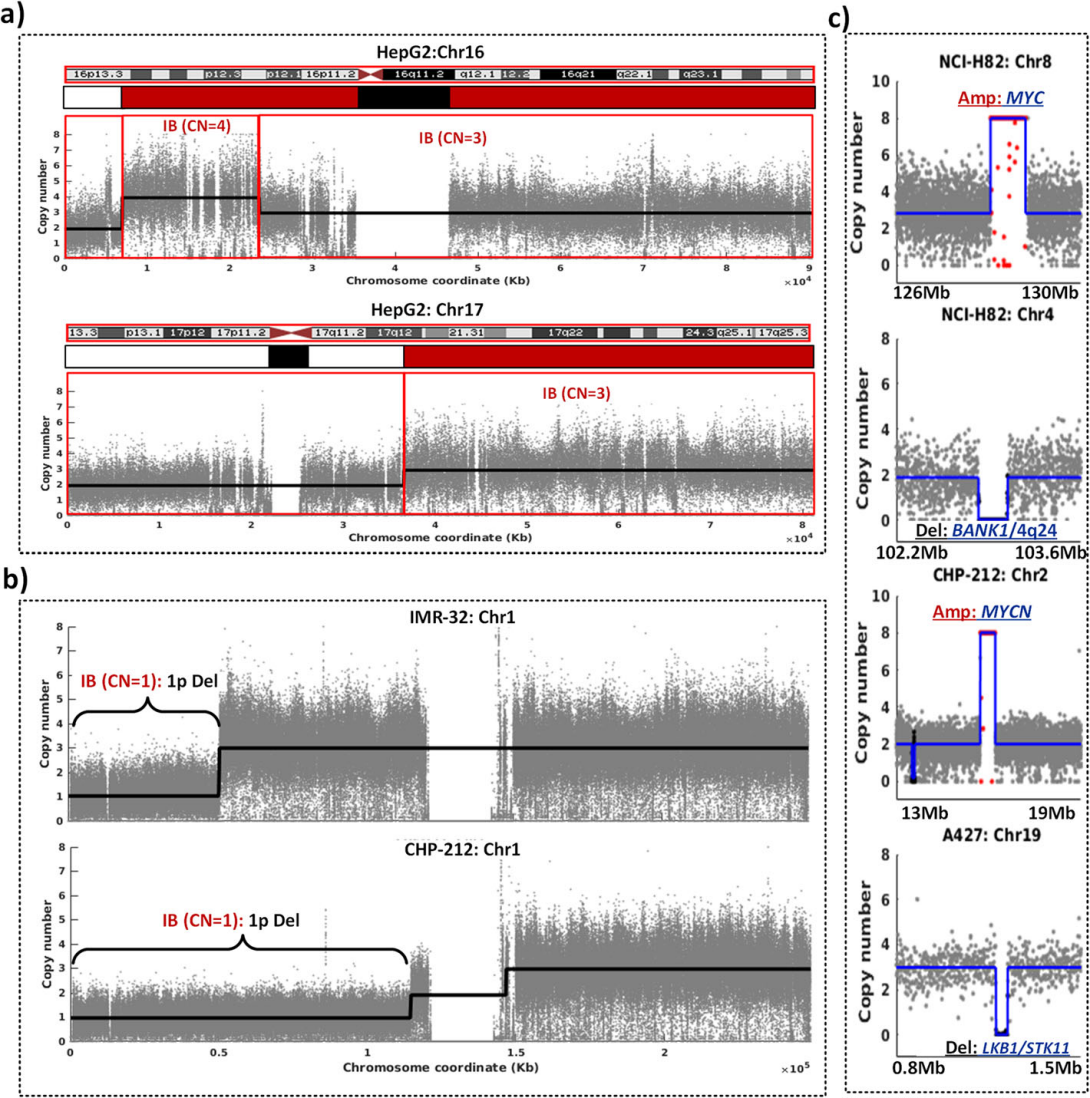
CNAtra validation of well-known LCVs and FAs. **a** CNAtra correctly detects the large segmental aneuploidies of Chr 16 (top) and Chr 17 (bottom) of HepG2 cell line described earlier using array CGH (shown as red bars below the idiograms). The black bar denotes the centromere region while the white bar represents CN-neutral (2N) region. **b** CNAtra identifies monoallelic 1p deletion in both IMR-32 and CHP-212 neuroblastoma cells. **c** CNAtra detects focal amplifications (*MYC* and *MYCN* loci) and focal deletions (*BANK1*/4q24 and *LKB1/STK11* loci) in respective cancer cell lines

We evaluated the ability of CNAtra to detect and distinguish both LCVs and FAs using validated data as ‘ground truth’ from multiple cancer cell line datasets. First, CNAtra confirmed the complete genetic profile of LCVs of HepG2 reported earlier using array CGH (comparative genomic hybridization) analysis [39]. CNAtra correctly identified the whole chromosome (2, 16 and 20) and segmental (involving chromosomes 6, 14 and 17) gains (Fig. 3a, Supplementary Figure S4). Second, CNAtra precisely detected the well-known monoallelic 1p deletion in neuroblastoma cell lines (IMR-32 and CHP-212) [40] (Fig. 3b). In both these cell lines, CNAtra called 1p deleted region as a single LCV event with correct CN estimation (CN = 1). Finally, CNAtra successfully detected the previously-reported focal amplifications of *MYC* (NCI-H82) [41] and *MYCN* (CHP-212, IMR-32) [42] loci as well as homozygous focal deletions of *BANK1/*4q24 (NCI-H82) [43], *LKB1*/*STK11* (A427) [44], and *p16INK4a*/*CDKN2A* (A427) [45] loci in respective cancer cell lines (Fig. 3c; Supplementary Figure S4).

### CNAtra confirms the SNP array-derived copy number profiles of cancer cell lines available from COSMIC and CCLE databases

In order to estimate our performance in a genome-wide manner, we used copy number amplification data of CHP-212 and NCI-H82 cells which are available from both CCLE (Cancer Cell Line Encyclopedia) [46] and COSMIC (Catalogue of Somatic Mutations in Cancer) [47] databases (Supplementary Table 3). Using SNP (single-nucleotide polymorphism) array data, these amplified regions were called by PICNIC software [48] in COSMIC database and by circular binary segmentation (CBS) [26] in CCLE database.

We applied CNAtra on the low-coverage NGS data of CHP-212 (1.4x) and NCI-H82 (0.31x) cancer cell lines and compared our calls with SNP-derived calls available from COSMIC and CCLE databases. In COSMIC data, the CNAs have been called in a gene-centered manner where each region is associated with one or more genes. COSMIC data have 6 and 13 amplified regions for the CHP-212 and NCI-H82 cells respectively. We found that out of 6 amplified regions in CHP-212, CNAtra identified two as FAs and other four regions inside a single LCV (Supplementary Figure S5a). Similarly, for NCI-H82, 8 out of 13 amplified regions have been detected as FAs, and 3 are embedded in two LCVs. In comparison, we found that CCLE database detected 11 and 99 amplified regions in CHP-212 and NCI-H82 cells respectively. In CHP-212, CNAtra detected 10 out of 11 amplified regions as 4 FAs and 3 LCVs, and the rest 3 amplified regions are embedded inside one LCV. Similarly, 78 out of 99 amplified regions were detected by CNAtra in NCI-H82. Out of 78, CNAtra identified 11 as FAs, 7 LCVs and rest 60 are part of 19 LCVs. This suggests that CCLE calls are segmenting LCVs into smaller CNA segments (Supplementary Figure S5b). Taken together, CNAtra calls intersected with 89.5% of COSMIC and 80% of CCLE calls. The discrepancy in CNA calling by COSMIC and CCLE databases versus CNAtra may be attributed to different experimental (SNP array versus NGS) and computational approaches of identifying copy number changes.

It is interesting to note that there are only two CHP-212 and four NCI-H82 calls that were commonly detected by both COSMIC and CCLE as consensus amplification regions suggesting poor concordance to identify CNA events. All these six consensus regions are detected by CNAtra. However, the number of consensus regions is scanty for a robust assessment of performance. Also, COSMIC and CCLE-detected copy number events do not distinguish between LCVs and FAs.

### CNAtra successfully identifies copy number profiles of cancer patient samples with variable purity

Apart from abnormal karyotype of cancer genome, tumor purity adversely affects the genomic analyses of patient samples. The proportion of non-cancer cell types in the tumor sample imparts a strong influence on the outcome and biological interpretation of copy number estimation. This problem is generally countered by using matched control samples by some computational tools for the purity estimation and copy number detection [49, 50]. The flagship cancer project, TCGA had initially set a minimum of 80% tumor purity as a quality threshold for inclusion of tumor samples in their study which was later changed to 60% [51]. Using 21 cancer types data, the median purity level of tumor samples available from TCGA is estimated to be around 75% [51].

We next applied CNAtra to two publicly-available WGS samples of cancer patients: Case#6 pancreatic neuroendocrine tumor (PanNET) and Case#7 B-cell follicular lymphoma (Supplementary Table 1) with tumor cellularity of 80 and 90% respectively. The copy number information for these two samples are also available from the same database which were identified by VarScan 2 [52], a computation tool which uses paired case-control sample for copy number detection. RD signals from tumor/cancer samples generally showed that majority of the genome is CN neutral resulting in a RD frequency distribution with a prominent peak at 2N which better fits our multimodal distribution with diploid assumption (Fig. 2a). Therefore, for patient samples, it is recommended to model the RD signal distributions using the diploid assumption.

For Case#6, CNAtra accurately detected all the chromosome-level aneuploidies (as detected by VarScan 2) using solely the WGS data of the cancer (case) sample (Supplementary Figure. S6a). For example, CNAtra identified the characteristic loss of heterozygosity (LOH) of chromosome 3 and 11 as well as the copy number gain of 17q region in Case#6 that are frequently observed in patients with PanNET [53, 54] (Supplementary Figure S6b). We also observed a focal gain affecting 6p22.2 in Case#6, which is a recurrent feature of PanNETs [55] (Supplementary Figure S6b). In Case#7, chromosome-level alterations were largely absent. However, we easily interpreted Case#7 to be a male subject as the X chromosome has only one copy in contrast to the Case#6 (female subject) who has two copies of X chromosome (Supplementary Figure S6a). Our evaluation with two cancer patient data suggests that CNAtra can be successfully applied to clinical samples with ≥80% purity.

To further evaluate the impact of tumor purity on CNAtra calls, we used the publicly-available simulated tumor datasets with purity ranging from 60 to 90% [https://www.yfish.org/data/singleclone_2x/]. CNAtra successfully detected all copy number ‘events’ correctly from samples with purity of 60, 70, 80 and 90% (Supplementary Table 4) as visually illustrated by the CNA profiles of chromosome 2 at different levels of purity (Supplementary Figure S7a). However, in some cases/events, tumor purity information can be used to correctly estimate the exact copy number values. The success of CNAtra to detect CNA events in tumor samples, without relying on matched control samples, can be attributed to the utilization of CN interval and CN state for detecting CNA (Supplementary Figure S7b).

### CNAtra is a superior tool for detecting large-scale and focal alterations

**Fig. 4:**
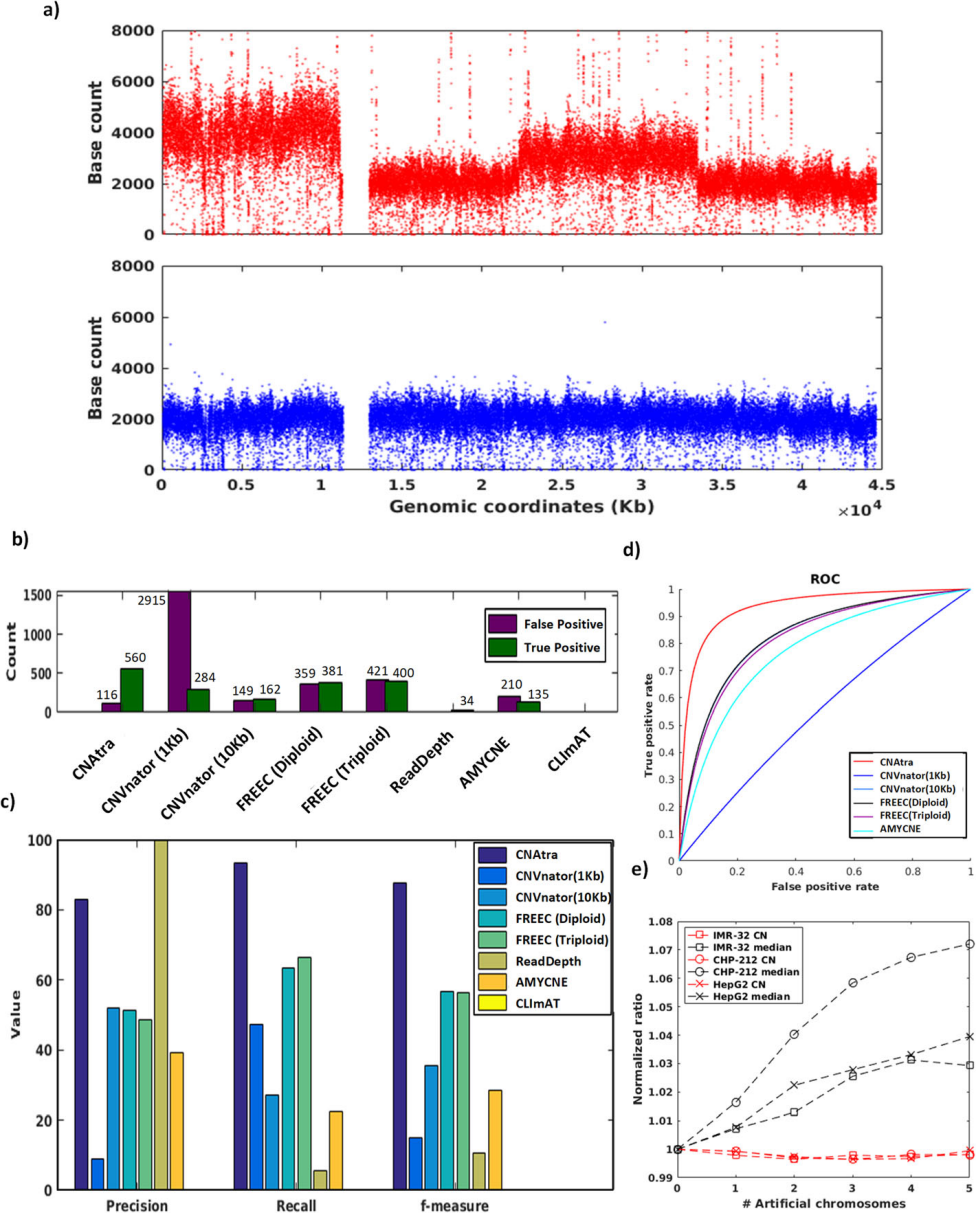
Performance evaluation of CN detection tools on the simulated datasets. **a** Coverage plot of simulated data containing spiked-in LCVs and FAs (top panel). Coverage plot of original CHP-212 Chr 12 (bottom panel) from which simulated data has been derived. **b** Bar graph showing false positive and true positive FAs detected by different tools. **c** Performance measurements (precision, recall and f-measure) of CNA detection tools for FAs. **d** Performance evaluation of FA calls by the ROC curves. **e** Line graph showing changes in CNAtra-estimated CN reference (dotted red line) and the global RD median (dotted black line) with each successive addition of artificial (simulated) chromosome in IMR-32 (square), CHP-212 (circle) and HepG2 (cross) datasets

In the absence of extensive experimentally-validated datasets of both LCVs and FAs, we used realistic simulated data where CNAs were artificially introduced to serve as ‘ground truths’ for performance evaluation. A simple simulation may not capture the inherent biases of the RD signal of cancer genomes. Therefore, we developed a novel approach to manipulate the original WGS reads of a cancer genome to randomly introduce FAs embedded within the LCVs maintaining the inherent features and complexities of the RD signal (Fig. 4a).

We generated simulated CNA profiles using low-coverage (<2x) CHP-212, IMR-32, and HepG2 data. We introduced LCVs and FAs in 5 chromosomes per cell line. For each chromosome, we have incorporated an average of 1–4 LCVs and then introduced 40 focal amplifications and deletions (size ranging from 50 to 100 kb) within these LCVs resulting in 600 FAs across three cell lines. Upon introducing the CNAs, the overall RD signal maintains the multimodal characteristics of the cancer genome (Supplementary Fig. S8a). Using this simulated data, we benchmarked the performance of CNAtra against five RD-based single-sample copy number detection tools which include ReadDepth [20], CNVnator [15], FREEC [16], CLImAT [22] and AMYCNE [17]. We analyzed the performance of these tools after optimizing their parameters for low-coverage data (see Extended Methods under Supplementary Information). We set the criteria of > 75% overlap between the spiked FA (ground truth) and the tool-estimated ‘call’ to be considered as a *true* call, and similarly, we set 90% overlap as the criteria for a *true* call in case of LCV. For a fair evaluation, false positives were estimated based on false amplifications only. This is due to the fact that many deletions may be falsely-identified at low-mappability regions as they are affected by the mappability thresholding method which varies between different tools. Our evaluation showed that CNAtra could overall detect 93.3% (560/600) of the ‘introduced’ FAs (IMR32 91.5%, CHP-212 97% and HepG2 91.5%) (Fig. 4b; Supplementary Table 5). In comparison, the second best tool, FREEC can detect FAs with 63.5% (381/600) accuracy under diploid assumption. All other tools (CNVnator, AMYCNE, ReadDepth, and CLImAT) can detect FAs with 0–50% accuracy (0 to 284 out of 600) (Supplementary Table 5). We also found that CNAtra has the highest accuracy for estimating CN correctly with average CN difference of 0.251N followed by CNVnator results with 10-kb binning (0.3642 N) and FREEC (0.4357 N) (Supplementary Figure S8b). To evaluate the detection power, we computed precision and recall (sensitivity) of each tool and found that CNAtra outperforms all other tools (Fig. 4c). Although ReadDepth showed the highest precision with zero false positives among all the tools, it can only detect 34/600 (5.6%) FAs constituting 33 deletions, and one amplification with wrong estimation of CN. Therefore, we compute f-measure to estimate detection accuracy which balances the precision and the recall values. CNAtra showed the highest f-measure value of 87.77% followed by FREEC with 56.8% (Fig. 4c). Next, we plot the receiver operating characteristic (ROC) curve for evaluating the performance of the tools. For estimation of true negatives and plotting the ROC curves only, we assume that the cumulative FA locus length in cancer cell lines is < 10% of the genome size. The ROC curve clearly shows that CNAtra is superior in detecting FAs in terms of true-positive and false-positive rates (Fig. 4d). When we compare the performance to detect LCVs, CNAtra again emerges as the best tool. CNAtra could detect 31/32 LCVs (96.8%) while CLImAT can detect 18/32 (56.25%) (Supplementary Table 5). Rest of the tools failed to detect any LCV event. We repeated this procedure to generate three additional simulated CNV profiles using different widths, frequencies and copy numbers. All the analysis showed similar relative performance between the tools as demonstrative using ROC curves (Supplementary Figure S8c; Supplementary Table 5).

In addition, we found that CNAtra is robust in estimating the CN reference regardless of the presence of LCVs. We stated earlier that LCVs could adversely affect local median that in turn can affect the CN estimation. For example, after spiking the RD signal with LCVs, the global median changes by 3–7% (Fig. 4e), which may lead to the wrong estimation of CN reference. Despite this, CNAtra can correctly estimate the CN reference (Fig. 4e). In addition, we also analysed the computation time of all single-sample CN detection tools (Supplementary Table 6). We have compared only the processing time for CN calling modules since different tools have different input formats. We found that for low-coverage datasets (<2x), CNAtra, ReadDepth and CLImAT take the shortest computation time (average 35, 45 and 41 s respectively).

### Visualization and manual inspection of CNA calls demonstrate CNAtra is best equipped to capture the complexity of cancer genomes

Review of CNA calls necessitates post-processing procedures which include visual inspection and curation of the results. Visualization and manual review of CNA profile in terms of copy number, size and structure can help to fine-tune the tool parameters as well as refinement and curation of the results for downstream applications. Therefore, CNAtra provides an interactive visualization platform for the user to inspect and authenticate its results manually. We utilized this visual inspection approach to comprehensively understand the advantages and limitations of all single-sample CN detection tools using cancer cell line datasets.

**Fig. 5:**
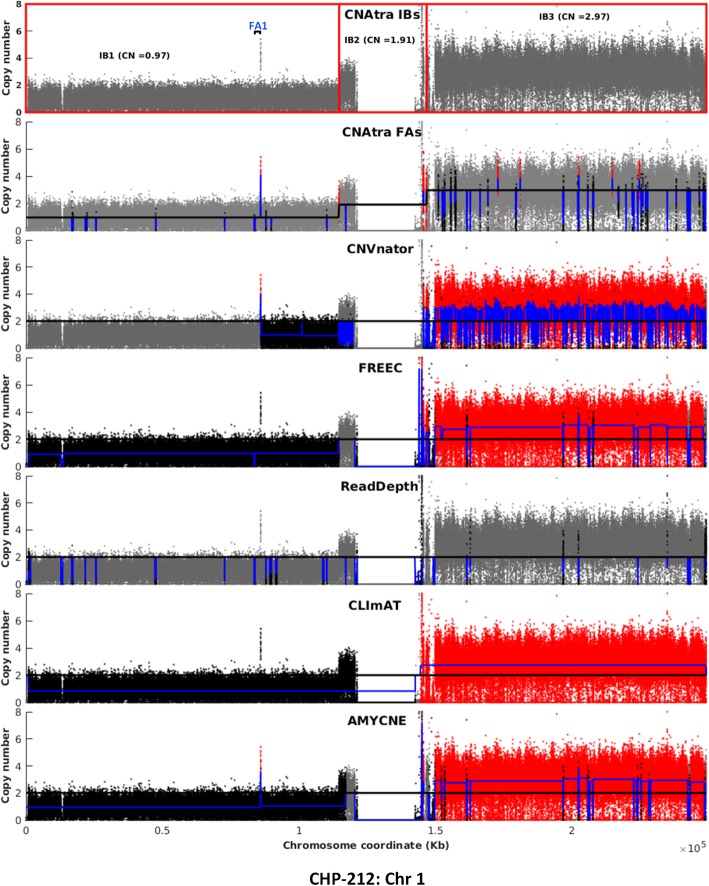
Visual comparison of CHP-212 Chr 1 CNA profiles generated by various single-sample CN detection tools. Red, black, gray dots are bins belonging to focally-amplified, focally-deleted and CN-neutral regions respectively. The blue line represents the copy number of each CNA. Any amplitude transition indicates a new CNA region. Top panel shows the IBs identified by CNAtra (each red box represents one IB). CNAtra examines each IB to detect the FAs (second panel). Rest of the panels show CNA output by other single-sample detection tools. IB1 is the monoallelic 1p deletion while FA1 is a focal amplification that is detected by CNAtra, CNVnator and AMYCNE

We found that CNAtra is the only tool to comprehensively detect both LCVs and FAs (Fig. 5; Supplementary Figures S9 and S10). Moreover, we found that most single-sample tools are affected by imperfect segmentation of the large segments. For example, all tools except CNAtra have wrongly divided the monoallelic 1p deleted region in CHP-212 neuroblastoma cells into several segments. Only CLImAT identified this 1p deletion as a single event; however, they fail to correctly determine the exact boundary of the segment (Fig. 5). Additionally, the focal amplification 1 **(**FA1**)** inside the monoallelic 1p segmental deletion, which harbors enhancer region based on Encyclopedia of DNA Elements (ENCODE) ChromHMM [56], is correctly detected by CNAtra, CNVnator and AMYCNE (Fig. 5). Also, 1q segmental amplification (correctly detected by CNAtra and CLImAT only) harbors many focal deletions. This confirms that focal amplification(s) can be a part of the monoallelic segmental deletion and similarly focal deletion(s) can be present inside a segmental amplification. None of the currently-available CN detection tools addressed the coexistence of LCVs and FAs in cancer genomes. Therefore, they cannot distinguish between these two phenomena and tend to favor the detection of either one of them. In addition, the estimated copy number is dependent on the proportion of LCVs in the genome for other single-sample tools. For example, ReadDepth wrongly estimated the 1p loss and 1q gain regions as CN-neutral regions in CHP-212 (Fig. 5). This effect is more evident in the A427 triploid cell line (Fig. 1d) [36]. As illustrated in the Supplementary Figure S9, IB2 with CN = 3 is misclassified as a CN-neutral region and IB3 with CN = 2 is wrongly identified as a deletion event, because the global median of the RD signal corresponds to the 3N state (black line in Fig. 1d, A427) and not the correct CN reference (2N) (blue line in Fig. 1d, A427). Moreover, all the tools are affected by overdispersion in low-coverage data which may result in false positives and false negatives as estimated using simulated data. CNAtra circumvents this problem using thresholding parameters. Additionally, the user can apply higher stringency thresholding to curate the CNA data manually.

## Discussion

Hyperploidy and pervasive genetic alterations are the hallmarks of cancer genomes. In the current study, we analyzed several cancer cell lines with different levels of aneuploidy which showed a complex multimodal distribution due to widespread LCVs and FAs. This is in stark contrast to unimodal RD signal distribution of normal human genomes (such as 1000 Genomes Project samples) which are mostly devoid of segmental aneuploidy. Current single-sample CN detection tools have limited ability to handle cancer genomes due to their assumption of unimodal probabilistic distributions of RD signal. Erroneous modeling of the RD signal distribution may lead to incorrect estimation of CN reference and false segmentation, which adversely affect the final CNA results. Consequently, cancer CNA profiles available from public databases (viz. COSMIC, CCLE) face the same problem. CNAtra successfully utilized a multimodal distribution to estimate the CN reference and then employed a CN-based merging algorithm to detect the large segments. Then, each CN-designated segment formed the basis for detecting FAs where the copy number of the parental segment is used as the local reference.

High-coverage (>15x) WGS datasets are generally used for CNA profiling; however, they are not available for many cancer cell lines. As an alternative solution, the NGS reads from ‘input control’ of histone modification ChIP-seq dataset, which are publicly available for many cell lines, can be effectively used for the same purpose. These input control reads are generated from sonicated crosslinked chromatin and are typically used for normalization and peak calling of the ChIP-seq data. These input data contain the genome-wide reads but they are generally sequenced at low-coverage (<2x). These data can be judiciously utilized to compute the RD signal for CNA analysis. However, any low-coverage dataset is afflicted with overdispersion of the RD signal which facilitates detection of a high number of false positives. CNAtra bypasses this problem by using coverage-based thresholding to detect high confidence FAs minimizing the number of false positives, which can be further tuned by the user for more stringent CNA calling. We have estimated the relationship between coverage and CNAtra parameters using subsampling of a limited number of high-coverage WGS datasets including one cancer cell line data. Our heuristic approach of determining coverage-based thresholding parameters leaves space for future improvements by incorporating additional high-coverage datasets. For example, the dispersion levels vary based on the CN state of each LCV. Therefore, training using additional datasets will provide better estimation of thresholding parameters at different CN states. Also, presence of poor-mappability regions (bins) can lead to false detection of homozygous focal deletions surrounding these bins. CNAtra provides the option to filter these focal deletions based on the percentage of low-mappability bins.

We have also successfully applied CNAtra to patient samples with variable tumor purity. Using simulated tumor data, we demonstrated the robustness of CNAtra calls across different tumor purity levels. Both these analyses were performed using only the test (case) samples, without utilizing any matched control sample. Generally, tumor contains an individual’s *germline* (inherited) CN variants as well as *somatic* copy number alterations (SCNAs). Distinguishing these two phenomena typically requires the paired case (tumor) and the ‘normal’ sample from the same individual. CNAtra uses a single sequencing sample at a time resulting in a CNA profile that contains both germline and somatic copy number variants. Nonetheless, we envisage that germline and somatic CN events can be effectively distinguished if both tumor and the matched normal sample are processed separately using CNAtra followed by comparative analysis of CN profiles from both samples.

One of the major limitations of the performance evaluation of CN detection tools is the non-availability of a complete repertoire of experimentally-validated cancer CN profile. Therefore, we generated realistic simulated data using the available cancer cell line data as input to maintain the signal variability and features of cancer RD signal. Then we randomly introduced CNAs of different length scales as ‘artificial’ ground truths by manipulating the short sequence reads in the binary alignment map (BAM) file. Read manipulation helps to capture the natural variations encountered in WGS data in an unsupervised manner. These simulated data with spiked-in CNAs can provide *de facto* ground truth for performance evaluation of CNA detection tools in terms of CN estimation, accuracy, and precision.

## Conclusions

CNAtra framework can detect and distinguish copy number changes from a single sequencing sample. The main advantages of CNAtra strategy can be summarized in three main points. First, CNAtra is the only tool to stratify LCVs and FAs which reflect important biological features. Second, multimodal modelling of RD signal helps to estimate absolute copy number in the absence of matched normal sample or SNP data. This empowers CNAtra to be applied to cancer cell lines and patient samples with a wide range of karyotype abnormalities. Finally, CNAtra can be applied to shallow-coverage WGS data. This will further allow the copy number discovery in many cancer cell lines for which ChIP-seq input control data are readily available from many epigenomic studies. To sum up, we believe that CNAtra is the ideal approach to model complex and low-coverage cancer datasets for detection of multi-level copy number changes. CNAtra has immense potential to add value towards the study of cancer genomes as well as discovery of novel CNAs.

## Methods

We explain the main modules of CNAtra framework (Supplementary Figure S11), coverage-based parameter calibration, and generation of simulated data in this section. The description of other modules of CNAtra pipeline and coverage-based parameter experiment is provided in Extended Methods under Supplementary Information.

### CNAtra framework

CNAtra is a MATLAB-based single-sample CNA discovery tool particularly adapted for low-coverage cancer genomes. CNAtra comprises two modules – RD calculator and CNA caller. In the RD calculator, we compute the RD signal as base count frequency at 1-kb bin from the input WGS data after initial read filtering steps. This allows us to fine-tune the tool to precisely define the boundaries for both LCVs and FAs in case of low-coverage data. We then correct the RD signal for systematic biases due to GC content (isochore normalization) and low-mappability regions. The CNA caller module constitutes the hierarchical framework to delineate the multi-level alterations in the cancer genome. We first compute the CN reference by fitting a multimodal distribution over the RD frequency histogram. Second, we use a multi-step framework to identify ‘large’ segments with distinct CN state. Third, we discover candidate peaks of ‘focal’ amplification and deletions in each CN-defined large segment.

#### Estimation of copy number reference

We utilize a multimodal distribution for computing CN reference (2N) and all other CN states (1N, 3N, 4N, …) based on the user-defined whole-genome ploidy level of a given input data (default = ‘free’). Our multimodal distribution is defined as the summation of normal distributions of different probabilities centered at different CN states (Fig. 2a):

$$ f(x)=\sum \limits_{i=2}^6c\ast {w}_i\ast \left(\frac{1}{\sqrt{\left(2\pi {\sigma}^2\right)}}\ast {e}^{-\frac{{\left(x-i\right)}^2}{2{\sigma}^2}}\right),x\ge 0 $$

such as

$$ {\int}_0^{\infty }f(x). dx=1 $$

$$ {\int}_0^{\infty}\sum \limits_{i=2}^6c\ast {w}_i\ast \left(\frac{1}{\sqrt{\left(2\pi {\sigma}^2\right)}}\ast {e}^{-\frac{{\left(x-i\right)}^2}{2{\sigma}^2}}\right). dx=1 $$

$$ \sum \limits_{i=2}^6c\ast {w}_i\ast {\int}_0^{\infty}\left(\frac{1}{\sqrt{\left(2\pi {\sigma}^2\right)}}\ast {e}^{-\frac{{\left(x-i\right)}^2}{2{\sigma}^2}}\right). dx=1 $$

$$ \sum \limits_{i=2}^6c\ast {w}_i=1 $$

$$ c=\frac{1}{\sum \limits_{i=2}^6{w}_i}, $$

where *i* is the CN state (2, 3, 4, 5, 6), *w*_*i*_ is the weight of the Gaussian distribution at state *i* and *c* is a constant for normalization of the probability distribution function. The “free model” assumes that all the CN states have the same weight (*w*_*i*_ = 1). For other models (‘diploid’, ‘triploid’, ‘tetraploid’), the weights *w*_*i*_ are 2^∣*i* − *n*∣^ where *i* is the CN state and *n* is the main ploidy state assuming that the majority of segments have CNs near this ploidy state.

CN reference is computed as the RD value that achieves the maximum overlap between the RD frequency distribution *r*(*x*) and the multimodal distribution *f*(*x*) of this ploidy assumption. These multimodal distributions work nicely for modeling RD signal of cancer cells as well as for normal cell lines which follow unimodal normal distribution since unimodal is a special case of the multimodal distribution (Fig. 1d).

#### Detection of candidate segments for IBs/FAs

We applied the Savitzky–Golay smoothing filter [57] to eliminate short-term variations and wave artifacts without affecting the ‘sharp’ signal change points. Savitzky-Golay filter is a method of data smoothing using a local least-squares polynomial approximation. Savitzky-Golay filter has advantages over other smoothing filters (such as moving average) since it tends to preserve features of the data such as sharp edges. Therefore, the RD signal can be smoothed without losing the locations of change points (Fig. 2a top panel**)**. Also, compared to the wavelet filter, Savitzky-Golay filter does not suffer from the shifting effect which is an essential characteristic to detect the accurate change points [58]. We then adapted the Modified Varri method [59] to detect the amplitude-shift points of the RD signal that define the boundary of primary segments. Combining Savitzky–Golay filter with Modified Varri segmentation enables the robust identification of true positives with low false discovery rate (see Extended Methods).

#### Identification of LCVs and FAs

A subsequent CN state-based merging process combines adjacent initial segments into large contiguous segments. We termed these merged contiguous segments with distinct CN state as IB. IBs with CN state different from CN reference are considered as segmental aneuploidy or LCVs. The size range of IBs (LCVs) are generally in the megabase (Mb) scale (default value ≥1 Mb, which can be tunable by the user). Each IB is used as a population of bins for the discovery of FAs within it. Assuming a normal distribution, we perform the t-test to identify the *statistically significant* FAs in each IB. Additionally, we define *high confidence* focal amplifications and deletions using coverage-based thresholding. These thresholds represent the minimum amplitude-shift between the estimated CN of candidate regions and their parent IBs to call FAs. We also filter out the FAs that are in blacklisted regions, gap regions, repeat-associated regions and low-mappability regions of the genome, or if they are smaller than CNAtra resolution. This resolution is the minimum width of FA that can be detected with false discovery rate (FDR) < 0.05 based on the genome coverage. The detailed explanation of IB assembly module as well as calling and filtering of focal alterations are provided in Extended Methods.

### Estimation of CNAtra calibration parameters

We used relatively high-coverage datasets (10x-14x), available from 1000 Genomes Projects (HG00119, HG01879, HG00096) and A427 cell line, for estimating the relationship between the data coverage and CNAtra parameters including resolution, amplification and deletion thresholds (Fig. 2d). For each dataset, we generated subsamples of the original data using Picard (http://broadinstitute.github.io/picard/) and SAMtools [60]. Then, we computed the optimum values of the analysis parameters of the original and subsamples (see Extended Methods). These values and their corresponding data coverages were used for fitting exponential regression models (Fig. 2d) which showed best-fitting compared to other regression models, such as polynomial, power decay and linear models (see Extended Methods).

### Generation of simulated copy number profile

We derived simulated CNA profile from real cancer cell line datasets. Starting from NGS reads (BAM file) of a cancer cell line data, we artificially introduced both LCVs and FAs of random copy number and width into cherry-picked chromosomes devoid of any observable large-scale copy number changes. The pipeline of artificial CNA generation contains two steps – 1) random selection of candidate location, and 2) artificial read spike-in. For each chromosome, we simulate *M* number of LCVs and *N* number of FAs. For this, we first divide the chromosome into *M* contiguous large segments randomly which represent LCVs. Similarly for focal alterations, we randomly choose *N* non-overlapping small regions (size range is user-tunable) which satisfy the following conditions: 1) regions must not overlap LCV boundary or any blacklisted/gap regions and 2) each region should have number of reads similar to the median of the selected chromosome based on threshold cut-off:

$$ \mathrm{Cut}-\mathrm{off}=\left(\raisebox{1ex}{$\mid {Med}_{LCV}-{RC}_{FAR}\mid $}\!\left/ \!\raisebox{-1ex}{${Med}_{LCV}$}\right.\right)\le 5\% $$

where *Med*_*LCV*_ is the median read count of the chromosome, and *RC*_*FAR*_ is the read count of the FA region.

After the selection of coordinates of LCVs and FAs, we next proceed for spike-in of artificial reads. In order to modify a selected region (*R*) with original copy number *C*1 to a new copy number *C*2, we add or remove $\left[X\ast \left(\frac{C2}{C1}-1\right)\right]$ artificial reads, where *X* is the initial number of reads in that region. These artificial reads were then spiked into the *R* region by randomly shifting the coordinates of the original reads by 10–500 bp. The original reads and the spiked-in artificial reads were merged into a new BAM file and used as input for CNA evaluation.

## Supplementary information

**Additional file 1. Supplementary Information containing Extended Methods and Supplementary Figures**

**Additional file 2.** Supplementary Table S1

**Additional file 3.** Supplementary Table S2

**Additional file 4.** Supplementary Table S3

**Additional file 5.** Supplementary Table S4

**Additional file 6.** Supplementary Table S5

**Additional file 7.** Supplementary Table S6

## Data Availability

Supplementary Information containing Extended Methods and Supplementary Figures as well as Supplementary Tables are provided as Additional files. All the datasets used in this study are publicly available (Supplementary Table 1). We used CNA profiles of cancer cell lines from CCLE [46] and COSMIC [47] databases for performance evaluation of CNAtra. The two WGS data (Case#6 and #7) of human tumor/cancer samples are obtained from Texas Cancer Research Biobank and Baylor College of Medicine Human Genome Sequencing Center.

## Abbreviations

BAM: Binary alignment map
CBS: Circular binary segmentation
CCLE: Cancer cell line encyclopedia
CGH: Comparative genomic hybridization
Chr: Chromosome
CN: Copy number
CNA: Copy number alteration
CNV: Copy number variation
COSMIC: Catalogue of somatic mutations in cancer
ENCODE: Encyclopedia of DNA elements
FA: Focal alteration
FDR: False discovery rate
IB: Iso-copy number block
LCV: Large-scale copy number variation
LOH: Loss of heterozygosity
NGS: Next-generation sequencing
PanNET: Pancreatic neuroendocrine tumor
RD: Read depth
ROC: Receiver operating characteristic
SCNA: Somatic copy number alteration
SNP: Single-nucleotide polymorphism
TCGA: The cancer genome atlas
WGS: Whole-genome sequencing
