# Supplementary material for: Hierarchical discovery of large-scale and focal copy number alterations in low-coverage cancer genomes
Source: BMC Bioinformatics. 2020 Apr 16;21:147. doi: 10.1186/s12859-020-3480-3 (PMC7160937; doi:10.1186/s12859-020-3480-3)
Supplement: Supplementary file 1 — Additional file 1. Supplementary Information containing Extended Methods and Supplementary Figures [file 12859_2020_3480_MOESM1_ESM.docx]

**Supplementary Information:**

**Hierarchical discovery of large-scale and focal copy number alterations in low-coverage cancer genomes**

Ahmed Ibrahim Samir Khalil^1^, Costerwell Khyriem^2^, Anupam Chattopadhyay^1,*^, Amartya Sanyal^2,*^

^1^School of Computer Science and Engineering, Nanyang Technological University, 50 Nanyang Avenue, Singapore 639798.

^2^School of Biological Sciences, Nanyang Technological University, 60 Nanyang Drive, Singapore 637551.

^*^Corresponding e-mail: anupam@ntu.edu.sg; asanyal@ntu.edu.sg

**EXTENDED METHODS**

**1. CNAtra Pipeline**

This section explains the CNAtra pipeline (Supplementary Fig. S11) in a stepwise manner.

***Binning the short reads***

CNAtra accepts BAM/SAM files as input sequence alignment data for RD calculation. It can handle single-end (SE), or paired-end (PE) reads. A MATLAB BioMap object is constructed from the input sequences to store the mapped short reads and compute the RD signal. These reads are filtered based on their mapping quality (MAPQ) to remove polymerase chain reaction (PCR)-duplicated reads, partially mapped PE reads and non-uniquely mapped reads. We also remove reads mapped to chromosome Y and chromosome M (mitochondrial genome) from the downstream analysis. Subsequently, reads that pass the filtering steps are used to calculate base (nucleotide) count at 1-kb non-overlapping bin (RD signal per bin). For SE reads, each read is considered as a sequencing fragment. For PE reads, sequencing fragment is reconstructed from the start of side 1 to the end of side 2. Genomic data coverage is computed as the ratio between the summation of lengths of these sequencing fragments and the genome size.

***Bin correction of the RD signal***

CNAtra corrects the RD signal for GC-content [[1](#_ENREF_1)] and mappability [[2](#_ENREF_2)] biases. Based on the read length, we compute the mappability score as the fraction of unique mappable nucleotides per bin. For that, we used the Umap tracks of uniquely-mappable regions [[2](#_ENREF_2)]. RD signal is first normalized for mappability effects. The base counts per bin are divided by the mappability score of that bin. Also, bins with low-mappability scores (< minimum mappability threshold) are filtered out to prevent overcorrection. User can set the minimum mappability threshold from 0 to 1 (default 0.5). Next, we correct the RD signal for GC-content using the CNVnator approach [[3](#_ENREF_3)]. GC ratio per bins can be calculated from the input reads, or they can be downloaded from the pre-computed GC-tracks (default option) for different read-lengths [[4](#_ENREF_4)].

***Estimation of copy number reference***

Based on the user-defined ploidy level (‘diploid’, ‘triploid’, ‘tetraploid’ or ‘free’), the copy number (CN) reference is defined as the RD value that achieves the maximum matching between the RD frequency distribution $r(x)$ and the multimodal distribution $f(x)$ of this ploidy assumption. For computing the CN reference of a given cell line, RD signal is re-binned at 100 kb to make the RD frequency distribution $r(x)$ centered around or near CN states. Then, the interval of RD values $[s, e]$ is divided into $m$ equally-spaced candidates of a CN reference. At each candidate value $i$, a multimodal distribution $f_{i}(x)$ is generated assuming that $i$ is the CN reference (corresponding to $x$ = 2 in the multimodal distribution). A rank $R_{i}$ is computed as the convolution between the RD frequency distribution $r(x)$ and the multimodal distribution $f(x)$:

$$R_{i}= \sum_{j=s}^{e} r(j)*f_{i}(j), i\in[s, e]$$

Finally, candidate value $i$ with the maximum rank $R_{i}$ is chosen as CN reference and used to normalize the RD-signal.

***RD segmentation***

We have developed our L1 segmentation procedure based on signal processing methods (Supplementary Fig. S12). First, the RD signal is smoothed to attenuate its fluctuation using the Savitzky-Golay filter [[5](#_ENREF_5)]. Then, it is oversegmented to capture all sharp-edge (change-points) by adapting the Modified Varri method [[6](#_ENREF_6)]. First, we find the peaks and troughs of the smoothed RD signal. Then, change points are computed as the mid-points between each consecutive peak and trough. Finally, the change points are selected based on target size of IBs and FAs. Our oversegmentation approach depends on the usage of both Saviztky-Golay filter and Modified-Varri segmentation together. To illustrate this, we applied CNAtra with and without smoothing (Savitzky-Golay filter) on the simulated datasets. Our results show that true positives decrease significantly in case of FAs in the absence of Savitzky-Golay filter (from 79-93% to 31-41% in 4 experiments using simulated datasets) while the numbers of LCVs remain unchanged (Supplementary Fig. S13).

We apply this procedure twice with two Savitzky-Golay frame lengths to identify the large and small segments. Large segments are chosen expecting one segment per 1 Mb region (the minimum IB width parameter) and used these large segments to build the iso-copy number blocks. Short segments are used to identify the FAs. They are selected expecting one segment per 10 times of CNAtra resolution, assuming that at least 90% of the genome is free of FAs.

***Assembly of iso-copy number blocks (IBs)***

We have developed the IB assembly module (Supplementary Fig. S14), a heuristic algorithm to build the IBs from the primary segments that resulted from the L1 segmentation procedure. We define the IB as a large segment (> user-defined minimum IB width) with distinct copy number from its adjacent segments, and it is not a part in a larger IB (>5% of its embedding IB). The primary segments are then classified based on segment width, segment copy number ($\mathrm{CN}_{\mathrm{segment}})$, and segment state/interval ($I_{\mathrm{segment}}$). First, subsequent primary segments with the same CN state are merged to form the initial segments (Supplementary Fig. S15 top panels). These initial segments are again merged iteratively to form the final IBs (Fig. 1b middle panel; Supplementary Fig. S15 bottom panels). In each iteration, long initial segments ($>$ the user-defined minimum IB width) are labeled first. Then consecutive long initial segments, with the same CN interval, are assigned to the same label. Then, if two long initial segments are interrupted by short initial segment(s), these short segments are assigned to a label of one of the surrounding long segments or to a new label to maximize intra-segment similarity based on their copy numbers.

***Calling of candidate FAs***

Focal alterations are detected by examining the candidate regions (L1 short segments) versus the IBs that are resulted from the IB assembly algorithm. We consider each IB as a population of bins and find its significant amplified/deleted regions. Each IB is used as a local reference for its candidate FA regions and modeled using a normal distribution. They are classified by the statistical t-test and the coverage-based thresholding test to find the initial significant FAs. Class 1 FAs pass the t-test while Class 2 FAs pass the thresholding test.

The t-test may result in many false positives for large regions especially in low-coverage data. Therefore, we integrated the random sampling approach to apply the t-test on segments of fixed reasonable sample size (*m*) and find the statistically significant candidates (Class 1 FAs). If the region size ($n$) is greater than the sample size (default = 35 bins), the t-test is applied on $k$ random samples and the maximum $P_{value}$ is chosen as the $P_{value}$of the region:

$$t_{\mathrm{region}}=\left\{ \begin{aligned} \max\left( \frac{\mathrm{CN}_{\mathrm{sample}_{i}\left( \mathrm{region} \right)}-\mathrm{CN}_{\mathrm{IB}}}{S_{\mathrm{sample}_{i}\left( \mathrm{region} \right)}}*\sqrt{m} \right) , i \in\left[ 1,k \right] if n>m, \\ \frac{\mathrm{CN}_{\mathrm{region}}-\mathrm{CN}_{\mathrm{IB}}}{S_{\mathrm{region}}}*\sqrt{n}, otherwise, \end{aligned} \right.$$

where $\mathrm{CN}$ is the estimated copy number, $S$ is the standard deviation, $m$ is the size of the sampled region. $P_{value}$ is then corrected for multiple hypotheses testing assuming that at least 90% of each IB are not FAs:

$$P_{\mathrm{value}}= \frac{P_{\mathrm{value}}\left( t_{\mathrm{region}} \right)*{Width}_{IB}*0.90}{{Width}_{region}},$$

$$statistical­condition=sign\left( \mathrm{abs}\left( P_{\mathrm{value}}-\alpha\right) \right),$$

where $\alpha$ is the threshold for a statistically significant region (default value α = 0.05). In addition, we use a thresholding method to define *high confidence* candidate regions (Class 2 FAs):

$$coverage­condition=sign\left( \mathrm{abs}\left( \mathrm{abs}\left( \mathrm{CN}_{\mathrm{region}}- \mathrm{CN}_{\mathrm{segment}} \right)-\lambda\right) \right),$$

where $\lambda$ is the coverage-based amplification or deletion threshold of highly significant regions. Then, adjacent significant regions of the same class and same type (amplification or deletion) are merged.

***Filtering the candidate focal alterations***

Candidate FAs are filtered based on coverage-based parameters and user-defined flags to keep only FAs with a high degree of confidence. First, we filtered out candidate FAs around the low-quality and low-abundance regions of the genome (dark bins). Low-quality regions are the bins which are located around centromeres, telomeres and blacklisted regions [[7](#_ENREF_7)]. Low-abundance regions are mostly associated with gap regions, repeated regions, and low-mappability regions. User can set a threshold of the maximum-allowed dark bins to retain a candidate FA (default = 0.5). In addition, we removed regions with small widths (less than the CNAtra resolution).

**2. Estimation of CNAtra tuning parameters**

We utilized the exponential regression models to estimate our coverage-based parameters including resolution, amplification and deletion thresholds (Fig. 2d). Resolution is defined as the minimum width of FAs that can be detected to achieve a low FDR ≤ 0.05. It is used as the threshold to filter the alteration regions at FA filtering step. Same resolution is also used as the Savitzky-Golay frame length to normalize the RD signal at L1 segmentation stage. Amplification and deletion thresholds are used to define the Class 2 focal amplification and deletion regions respectively (Fig. 2d). These thresholds are the minimum differences between the copy number of amplified/deleted regions and the copy number of their IB.

For the resolution analysis, at each genomic coverage ($x$), 1-kb binned RD signal was first modeled using a normal distribution $(\mu,\sigma)$. Assuming that maximally 10 % of the genomic regions can be FAs, we generated random RD values for 5%, 90%, and 5% of bins from normal distributions corresponding to copy numbers of 1N, 2N, and 3N respectively (Supplementary Fig. S16). FDR was computed as the fraction of false CN-assigned bins to the total number of bins. We repeated these steps by incrementing the bin size. Resolution is computed as the minimum bin-size that achieves FDR of ≤ 0.05.

For the focal amplification (or deletion) thresholding studies, we used approximately 0.5N as the amplification (or deletion) threshold of the original high-coverage dataset, and we computed the average amplitude shift between the CN of the amplification (or deletion) region and the IB (reference amplitude shift). Then, for each subsample, we computed the new average amplitude shift between the copy numbers of these regions and the IB copy number (sample amplitude shift). The amplification (or deletion) threshold was adjusted by adding the difference between the sample amplitude shift and the reference amplitude shift.

The reason to utilize exponential regression models for estimating coverage-based parameters is as follows. We have tested exponential regression model as well as other regression models for modeling the CNAtra parameters. For that, we used MATLAB ‘fit’ function with different underlying models (‘ploy2’: $Y = p1*x^{2}+p2*x+p3$, ‘weibull’: $Y = a*b*x^{b-1}*e^{-a*x^{b}}$, ‘exp2’: $Y = a*e^{bx}+c*e^{dx}$, ‘power2’: $Y = a*x^{b}+c$). Then, we applied the two-sample Kolmogorov-Smirnov test (KS test) to validate each model assumption. Among these models, the KS test for both exponential regression model ‘exp2’ and power-decay model ‘power2’ didn’t reject the Null-hypothesis with asymptotic p-value > 80% for all the parameters. Therefore, theoretically both can be used for modeling the relationship between the data coverage and parameters. However, based on the manual inspection, we found that exponential model shows better fitting (Supplementary Fig. S17)

**3. Benchmarking experiment**

We generated four versions of the simulated datasets with different widths, frequencies, and copy numbers (Supplementary Table 5). The first dataset is generated with 40 FAs of 50-100 kb width per chromosome, and a minimum difference between LCV and FA copy numbers (CN shift) of 2N. This dataset is used for the detailed evaluation of performance for each tool. The second dataset is created with 30 FAs of 50-200 kb width per chromosome and CN shift of 2N. The third dataset is generated with 30 FAs of 50-200 kb width per chromosome and CN shift of 1N. The fourth dataset is generated with 40 FAs of 50-100 kb width per chromosome and CN shift of 1N. Using these artificial datasets, we performed comparative evaluation of the performance of CNAtra, ReadDepth, CNVnator, FREEC, CLImAT and AMYCNE. We could not pursue XCAVATOR [[8](#_ENREF_8)] and SCNV [[9](#_ENREF_9)] due to their complicated installation procedure. We optimized the performance of each tool to handle the overdispersion and multimodality of the low-coverage cancer datasets.

We found that CNVnator results change dramatically with the input bin size. At 1-kb binning, CNVnator detects many false positives. At 10-kb binning, false positives are decreased significantly. However, it misses some true focal alterations (true positives) that are detected using the 1-kb resolution as illustrated in Supplementary Figure S18. Therefore, we generated CNVnator results using bin sizes of 1-kb as well as 10-kb. The main parameter of FREEC is the whole-genome ploidy level of the cell line. For example, the organization of segments differs considerably at different ploidy levels as illustrated using the 12p region of NCI-H82 (Supplementary Fig. S19**)**. It uses the ploidy information to set the CN reference (2N) based on the median of the RD signal. This affects the segmentation and calling of CNAs. However, this cannot compute the CN reference accurately for hyperdiploid, near-triploid or higher ploidy cells. For example, median RD signal cannot capture any peak of the multimodal distribution for NCI-H82, MCF7, and LNCaP cell lines (Fig. 1d). We generated FREEC results of the artificial datasets under diploid and triploid assumptions for comparison. For CLImAT, we tried smaller values of the minDepth parameter (default 10) to adapt to the low-coverage data. We found that minDepth of 0.1 gives the best segmentation and calling results. For ReadDepth, there are many tuning parameters such as overdispersion (default 3), percCNGain (default 0.05), and percCNLoss (default 0.05). We tried higher values of overdispersion. Also, we set the percCNGain and percCNLoss based on the multimodal distribution of artificial data (Supplementary Fig. S8a). However, the results did not show notable enhancement. We believed that ReadDepth failed to model the multimodal distribution and to correctly estimate the CN reference. Therefore, most of their identified segments cannot be called as alteration events (e.g. 1p deletion and 1q amplification in Fig. 5). For AMYCNE, we use its default parameters since results showed a balance between true positives and false positives.

**SUPPLEMENTARY FIGURES**

**Figure S1**

**Profiling of normal and cancer cells in terms of coverage plot and statistical tools.** **(a)** Genome-wide coverage plot of 1000 Genomes Project samples (i. HG00119 and ii. HG00096). **(b)** Genome-wide coverage plot of cancer cell lines (i. HepG2 and ii. IMR-32). **(c, d)** Bar chart showing statistical measures (mean and median) of RD signal of 1000 Genomes Project samples **(c)** and cancer cell lines **(d)** along with CN reference estimated by CNAtra.


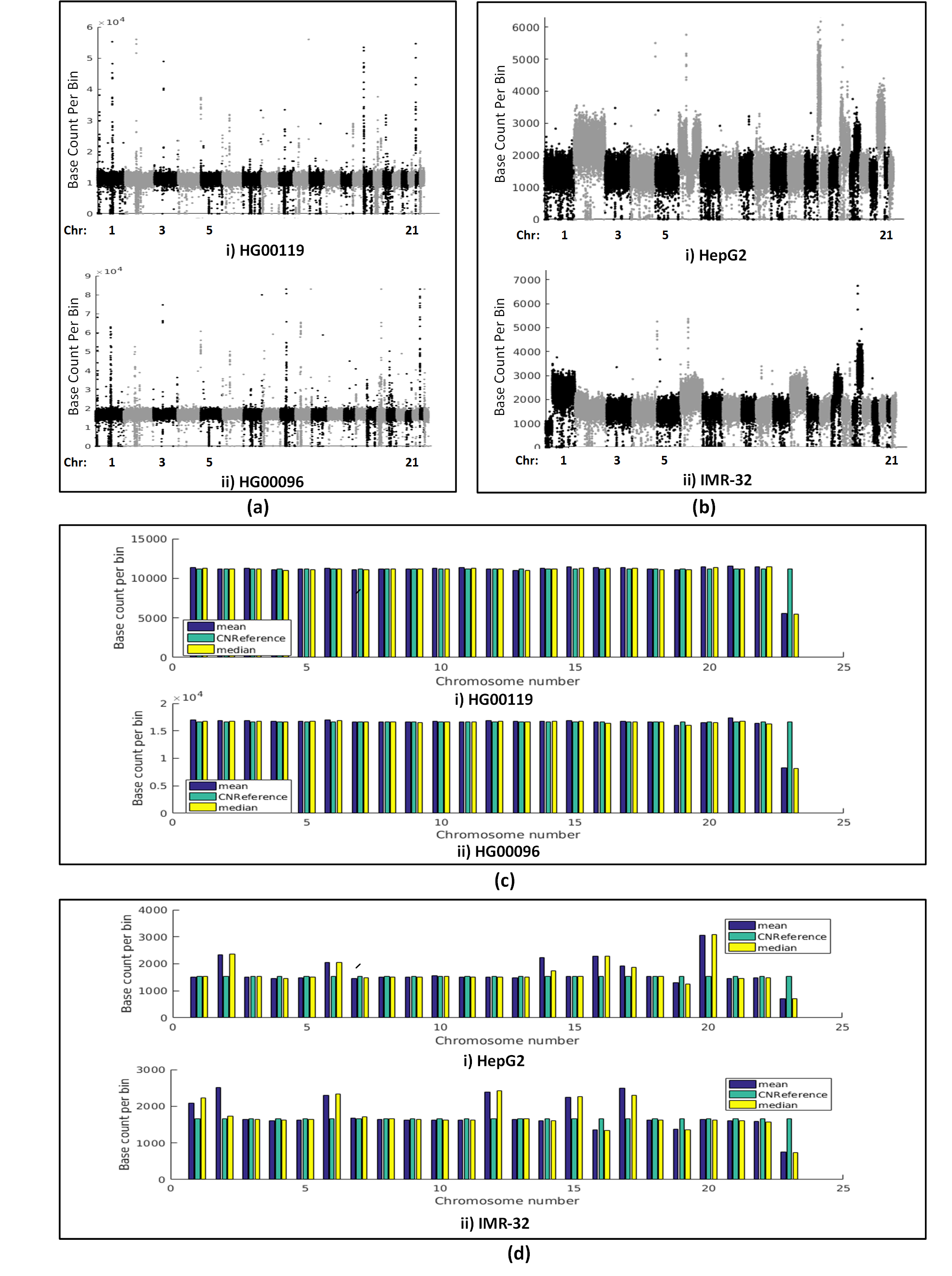


**Figure S2**

**Normality test for individual IBs using Q-Q plot and Kolmogorov–Smirnov test.** In each iteration, 1-Mb continuous sample from an IB is randomly selected, and Q-Q plots are generated for A427 **(a)**, CHP-212 **(b)**, HepG2 **(c)**, IMR-32 **(d)** and NCI-H82 **(e)** cell lines. P-values are calculated using the Kolmogorov-Smirnov test and indicated on top of each Q-Q plot.


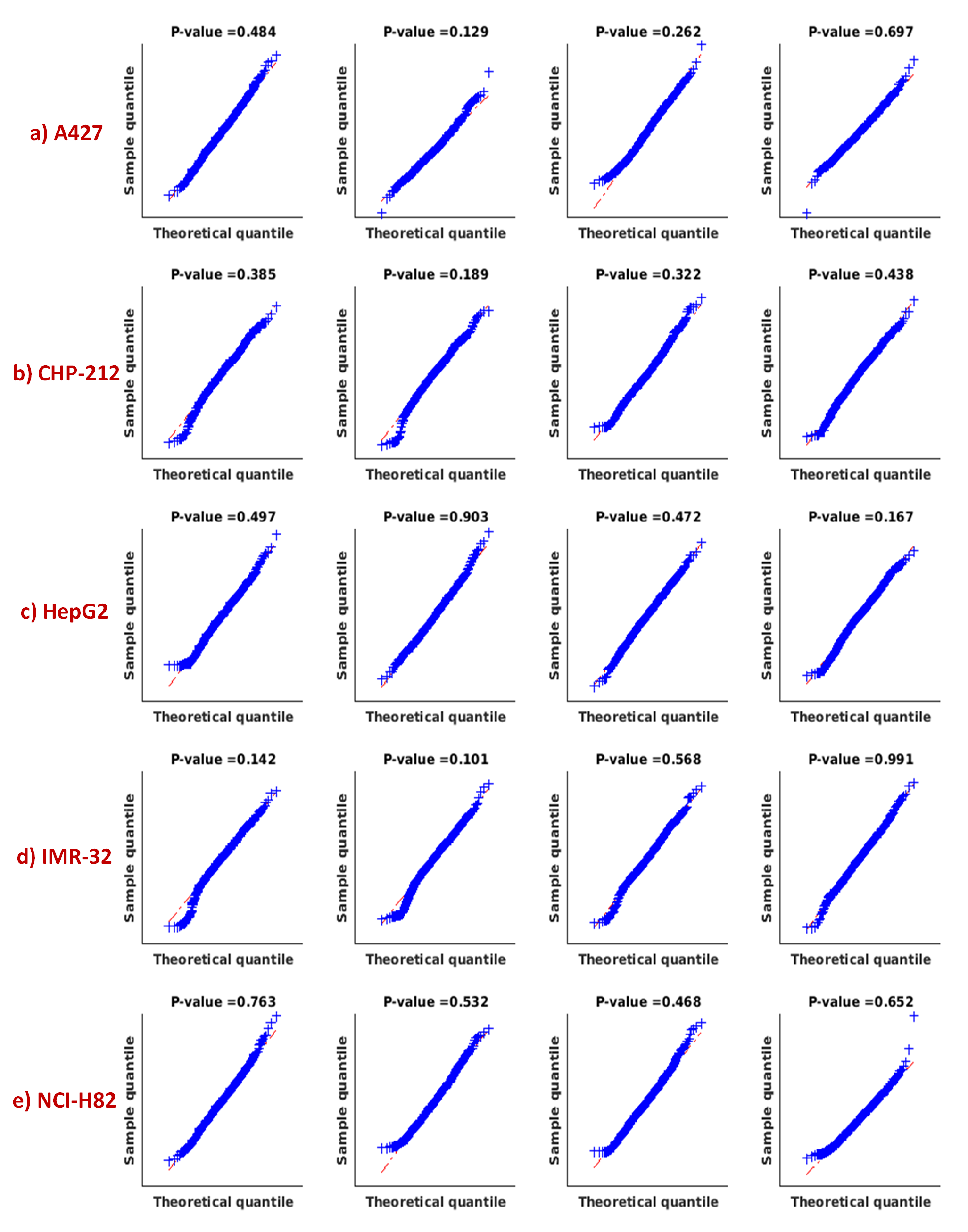


**Figure S3**

**Schematic representation of the expected coverage plot profile of a hypothetical diploid cancer cell line with focal alterations**. Theoretically, any region that is 0.5N shift from the 2N state may be considered as focal amplification (red dot) or deletion (gray dot). However, some of these regions may result from wave artifacts and overdispersion due to low-coverage data, such as FA4.

**
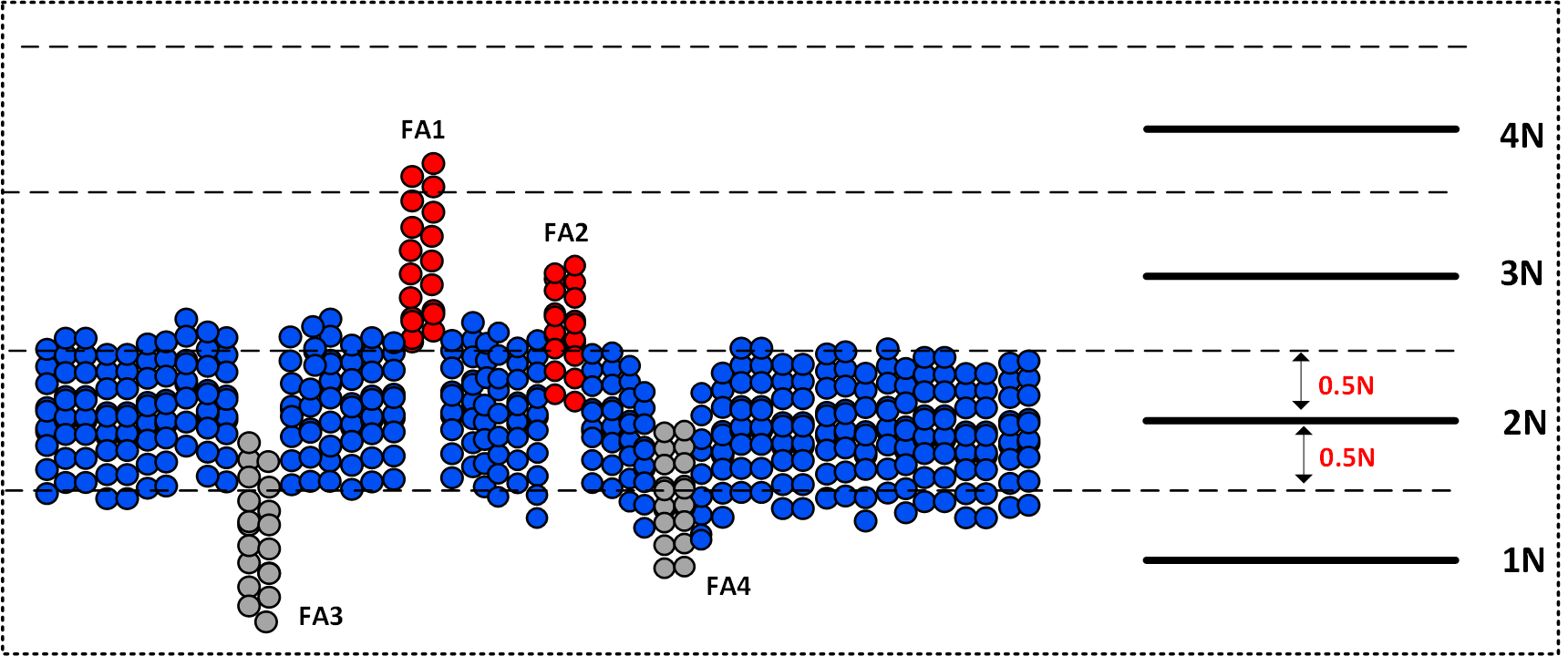
**

**Figure S4**

**Validation of additional LCVs and FAs by CNAtra in different cancer cell lines.** **(a)** CNAtra detected the previously-reported segmental aneuploidies in Chr 2, 6, 14 and 20 of HepG2 cell line using array CGH data. The bars below the ideogram indicate the segmental amplification (red), CN-neutral (white) and centromere (black) regions. CNAtra detected additional IB6 in Chr 14 using WGS data of HepG2. This difference can be attributed either to real biological difference between sublines/strains and passages of HepG2 used for the two experiments or difference in the experimental procedures (arrayCGH versus NGS-based detection). **(b)** CNAtra detects focal amplification in IMR-32 and focal deletion in A427 cell lines.

**
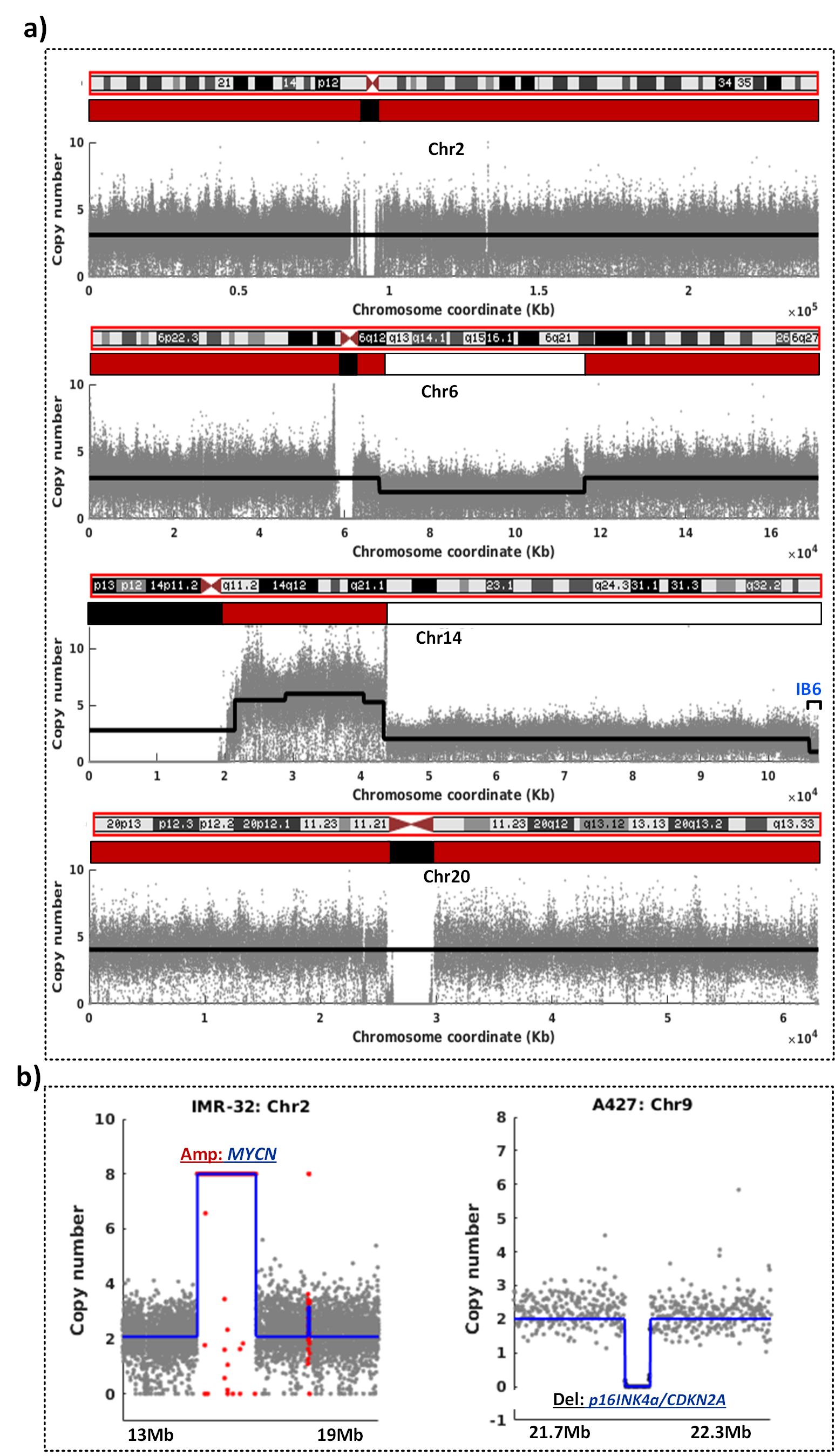
**

**Figure S5**

**Visual illustration of COSMIC and CCLE CNA calls (using SNP arrays) superimposed on the NGS-derived coverage plot. (a)** COSMIC calls are gene-centered with four CNAs of same copy numbers embedded in one IB (detected by CNAtra) in Chr17 of CHP-212. **(b)** CCLE calls divide NCI-H82 Chr 3q segmental amplification into many smaller CNAs. CNAtra detects the same 3q region as a single IB. Red, black and gray dots are bins belonging to focally-amplified, focally-deleted and CN-neutral regions. The blue line represents the copy number of CNA where any amplitude transition indicates a new CNA region.

**
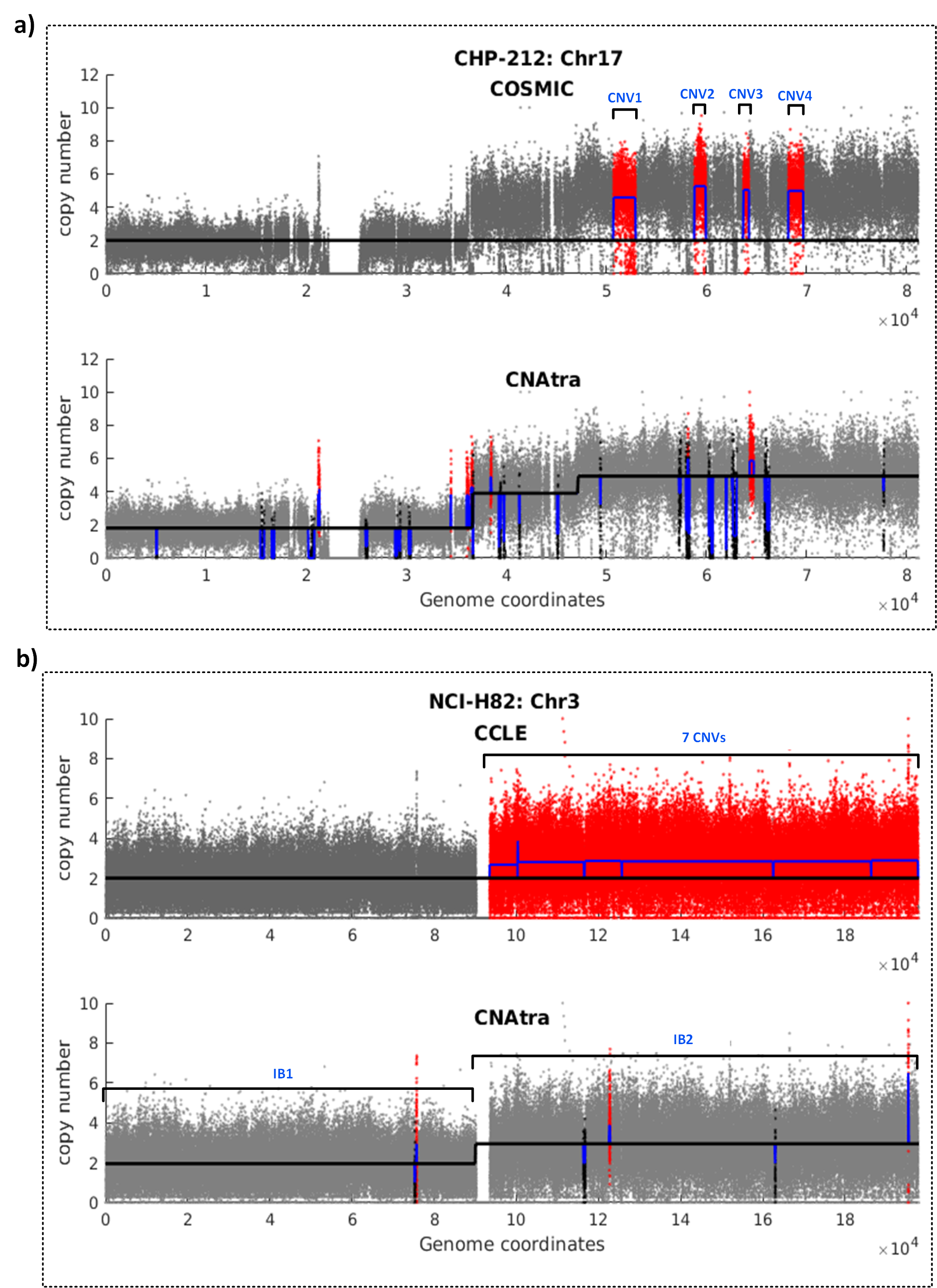
**

**Figure S6**

**CNAtra CNA profile using cancer patient samples. (a)** CNAtra detection of genome-wide CNAs in Case#6 (top) with PanNET and Case#7 (bottom) with B-cell follicular lymphoma with tumor cellularity of 80% and 90% respectively. **(b)** CNAtra detects focal gain affecting 6p22.2 (top), segmental loss of 15q region (middle) and segmental gain of 17q region (bottom) in Case#6 patient with PanNET.

**
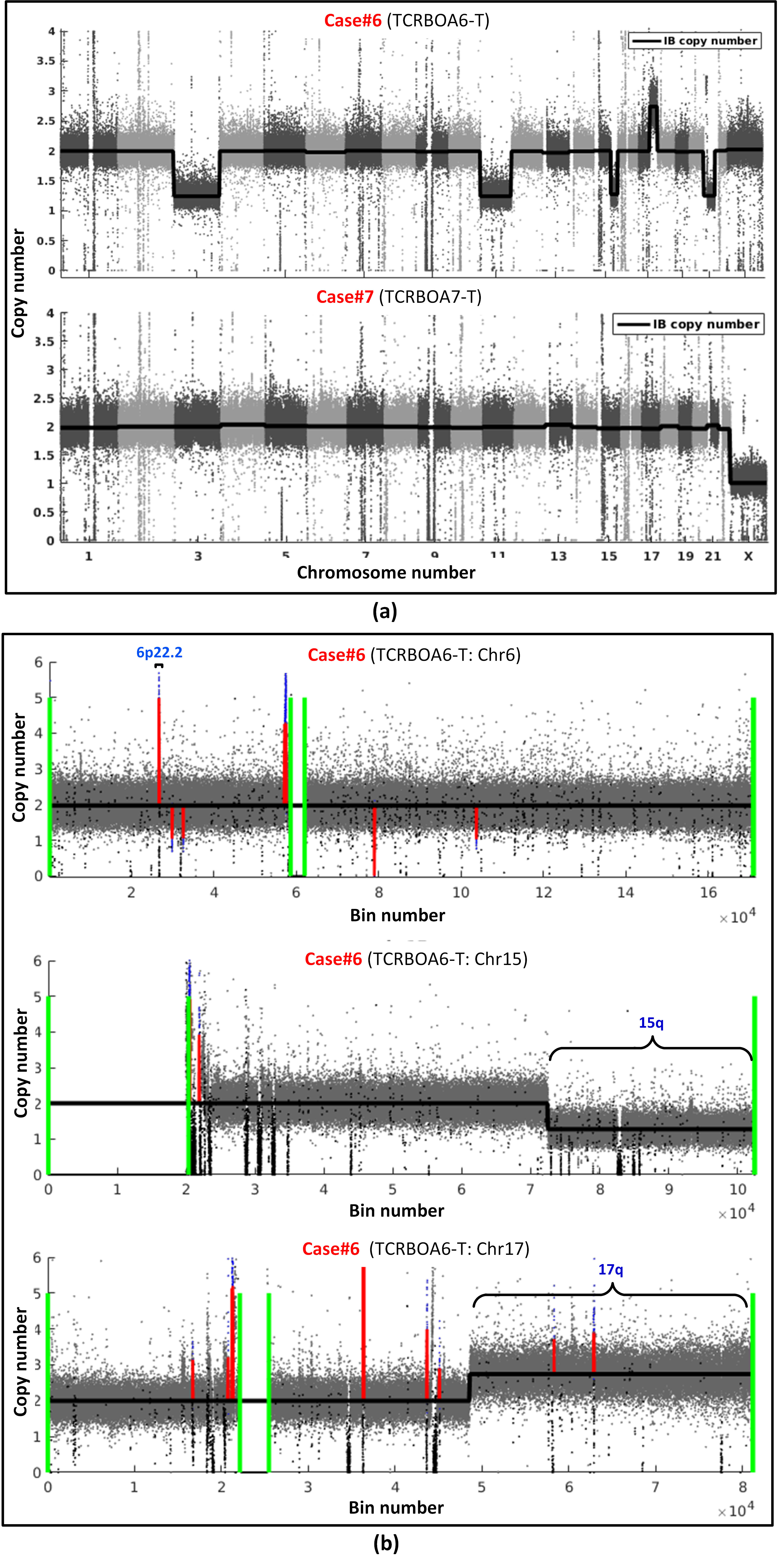
**

**Figure S7**

**CNAtra successfully identified the copy number events of simulated tumor data with purity >50%. (a)** Using the simulated tumor data of variable purity (<https://www.yfish.org/data/singleclone_2x/>), CNAtra detected all copy number ‘events’ correctly from samples with purity levels of 60% to 90%. This is visually illustrated by the CNA profile of Chr 2 at different purity levels. However, the estimated copy numbers of these events may need to be corrected in some cases based on the purity of the sample. **(b)** A schematic explanation of CNAtra calls in a hypothetical sample with tumor purity of 60%. For example, the copy number values of the red segment (originally CN = 1N in tumor cells) and blue segment (originally CN = 3N in tumor cells) will change to 1.4 and 2.6 respectively due to the ‘contamination’ of tumor sample with 40% non-cancerous (normal) cells. CNAtra can estimate their copy number correctly as 1N and 3N respectively based on the ‘interval’ definition of CN state. For example, 1N state ranges from copy number values of 0.5 to 1.5, and 3N state ranges from 2.5 to 3.5. This allows CNAtra to accurately estimate the CN-state changes up to 1N with tumor purity >50%, up to 2N change with purity >75%, and up to 3N change with purity >90%.

**
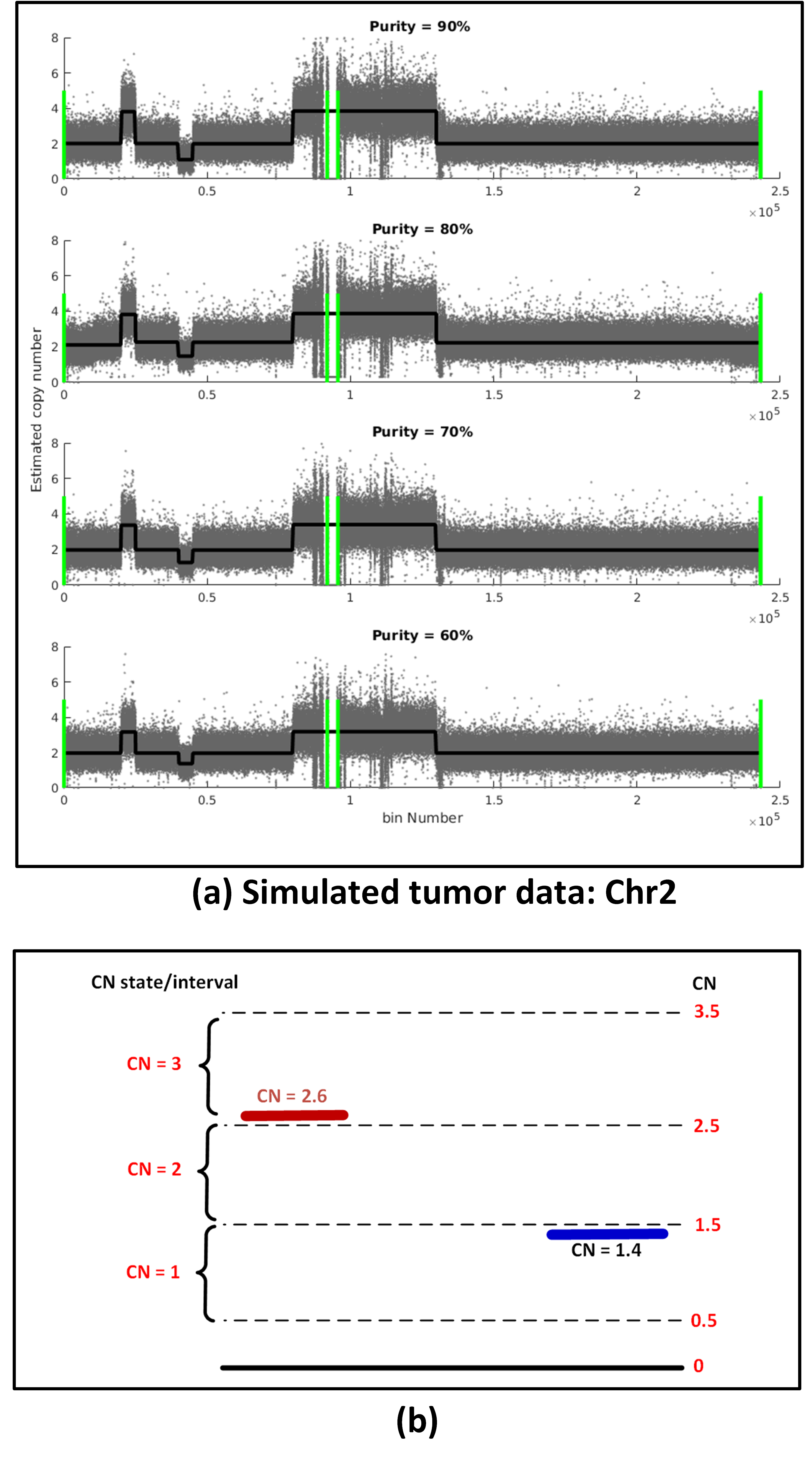
**

**Figure S8**

**Performance evaluation using simulated data. (a)** RD frequency distribution of IMR-32, CHP-212 and HepG2 cell lines after random introduction of LCVs and FAs in five chromosomes of each cell line (simulated data). The blue and black lines represent CN reference (2N) estimated by CNAtra and the global median RD signal respectively. The dotted blue line denotes the higher CN states (3N, 4N, 5N) based on CNAtra estimation of CN reference. **(b)** Box plot showing overlapping ratio between the spiked FAs and the CNA calls of each tool (top panel). The bottom panel shows the mean CN difference between the spiked FAs of known CN and the estimated CN of CNA calls of each tool. **(c)**  ROC curves for different detection tools obtained using similar evaluation strategy as described in Fig. 4d with different parameter settings (width, frequency and CN of the spiked FAs).

**
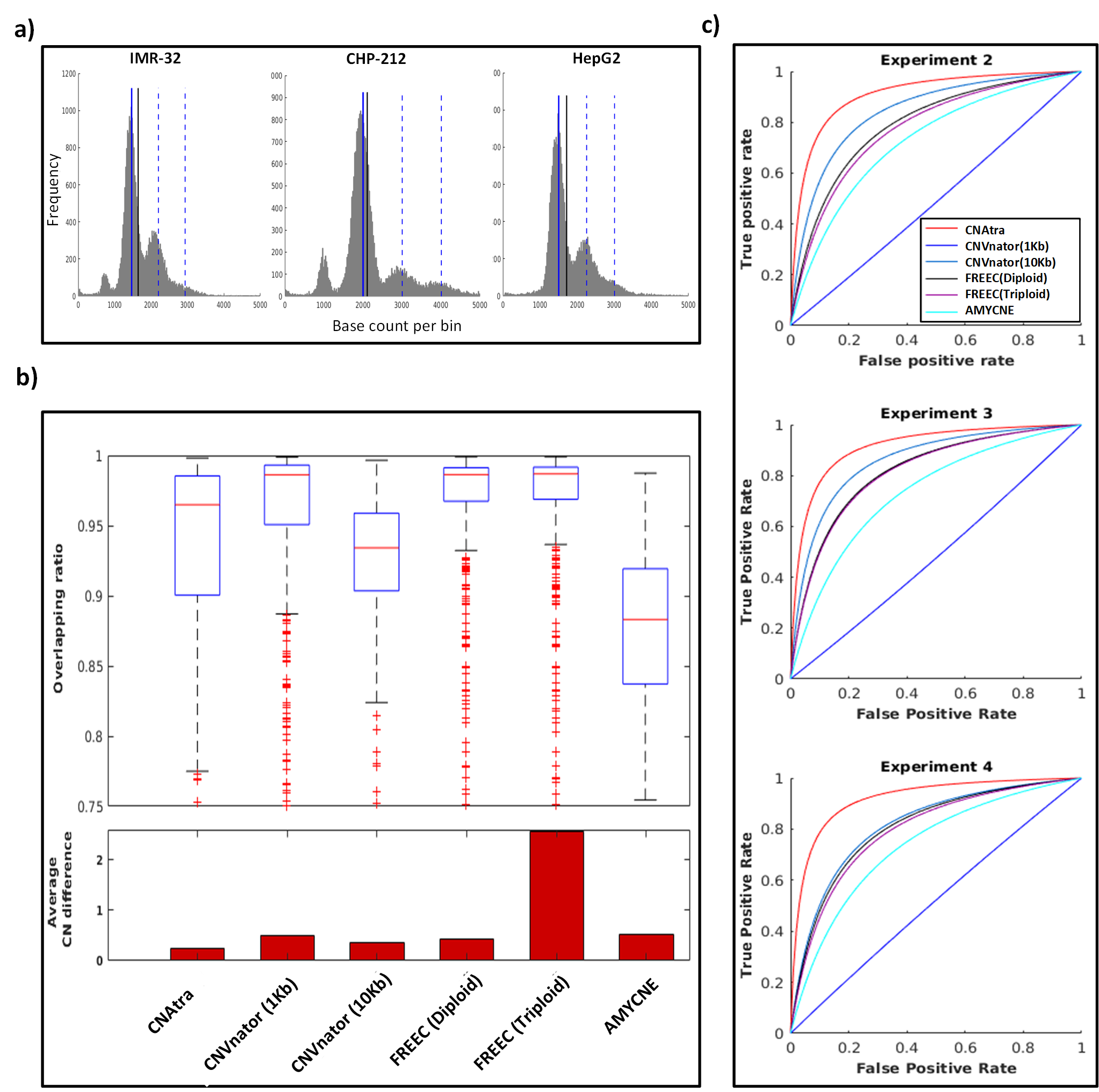
**

**Figure S9**

**Inaccurate estimation of segment copy number by currently-available tools in the absence of ploidy information.** Visual inspection of CNA results from Chr 3 (0-89 Mb) region of A427 (triploid) cell line showed that copy number is wrongly estimated for IB2 and IB3 by all the tools (except CNAtra), since these tools rely on the global median for estimating CN reference (2N).


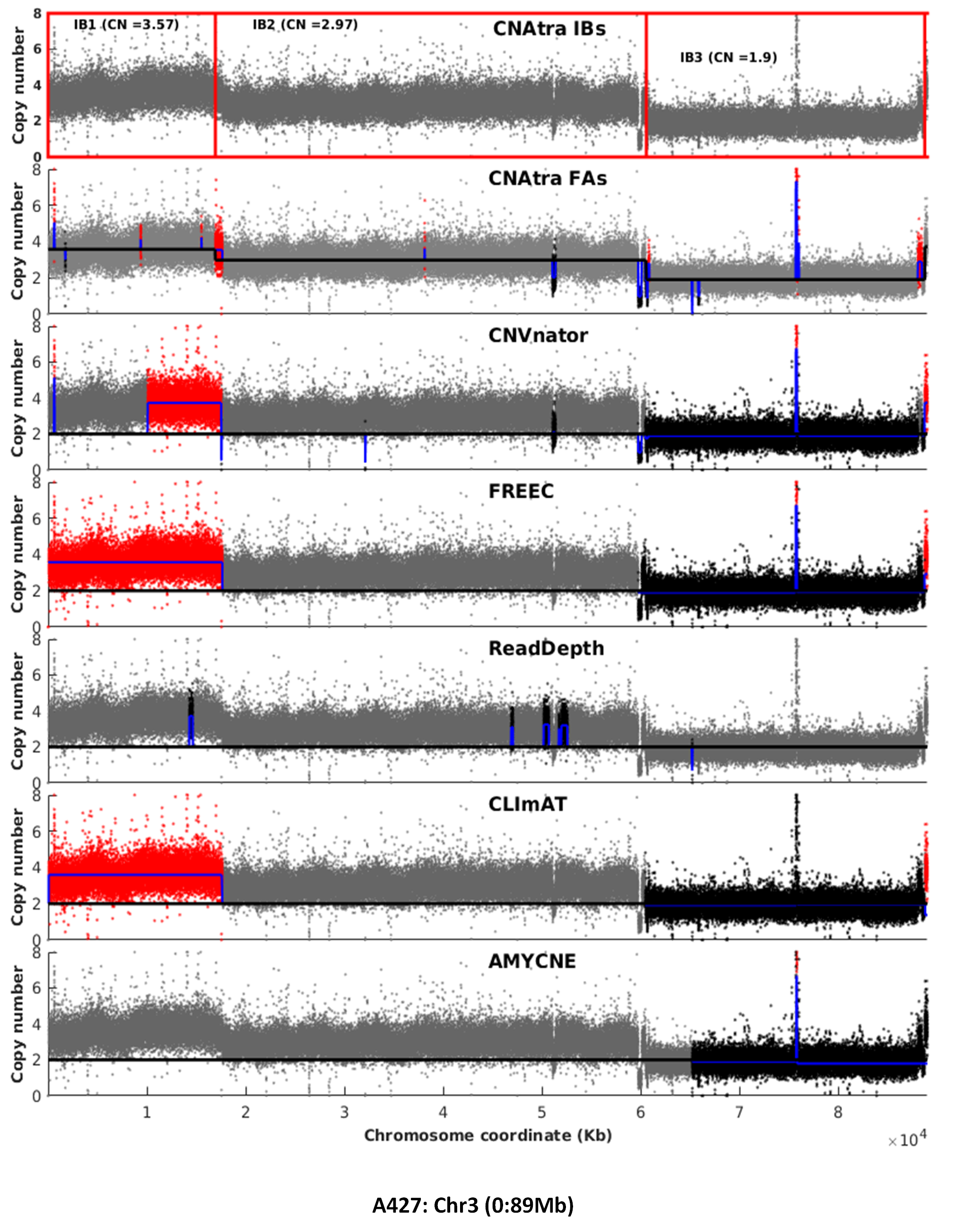


**Figure S10**

**Visual comparison of CNA profiles generated by CNAtra and other tools.** Manual inspection of CNA results from Chr 12 of NCI-H82 (0.31x coverage) showed that CNAtra approach is robust and unaffected by overdispersion and wave artifacts. Other CNA detection tools are adversely affected by signal variation resulting in false segmentation. Red, black and gray dots are bins belonging to focally-amplified, focally-deleted and CN-neutral regions. The blue line represents the copy number of each CNA where any amplitude transition indicates a new CNA region.


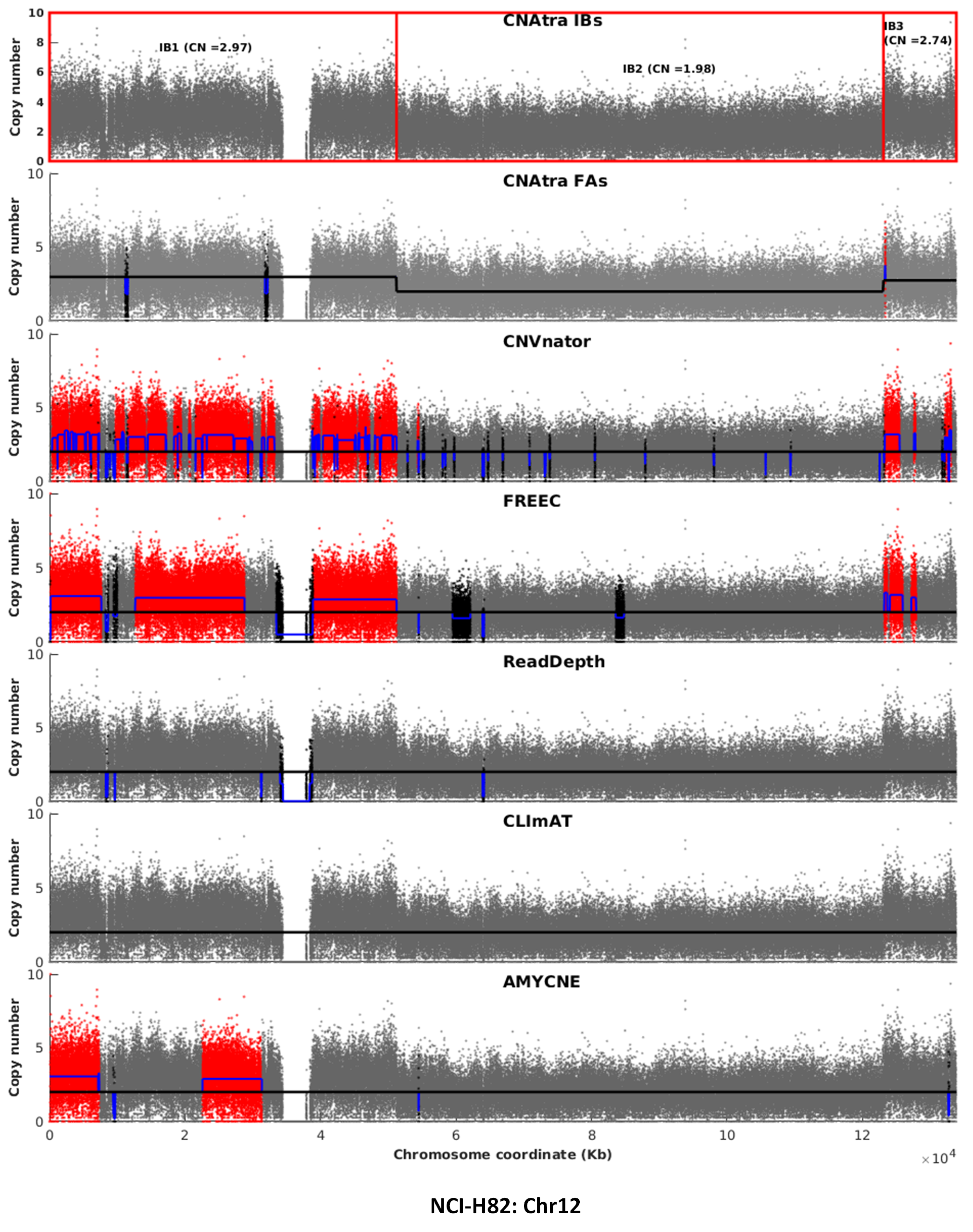


**Figure S11**

**Flowchart of the CNAtra pipeline**.


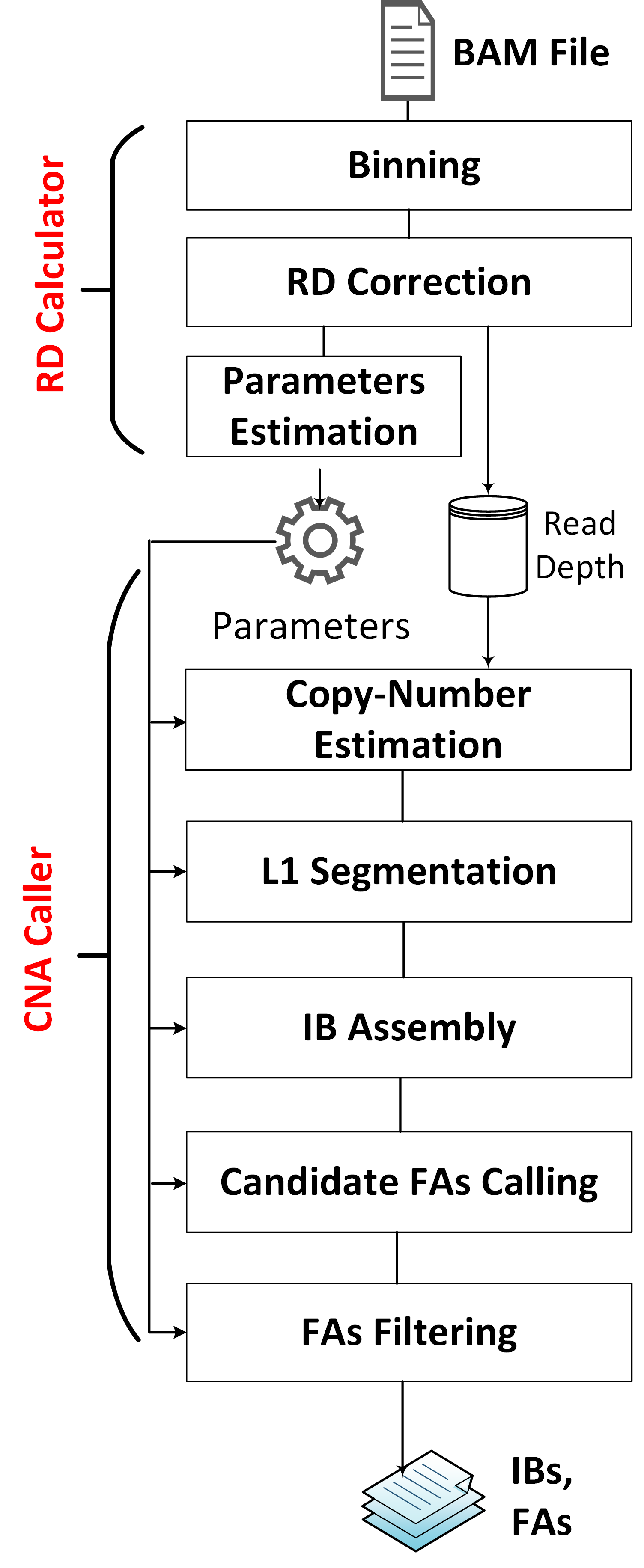


**Figure S12**

**Pseudocode for L1 segmentation.**

| **L1 Segmentation Procedure** |
| --- |
| Input: **F**, RD signal of a chromosome  Output: **E,** change points (starting and ending points of segments).   1. Smooth the RD signal using Savitzky-Golay Filter with window length (w):   ***F = SG_Filter(F, k=7, w)***  ***F = SG_Filter(F, k=1, w)***   1. Find the location of the peaks (X) and bottoms (Y) of the signal F. 2. Find the corresponding RD values of X and Y:   $\boldsymbol{F}_{\boldsymbol{x}}$***= F(X)***  $\boldsymbol{F}_{\boldsymbol{y}}$***= F(Y)***   1. Merge and sort the peaks and bottoms:   ***Z = [X, Y]***  ***F_z_ = [F_x_, F_y_]***  ***[Z. I ] = sort (Z)***  ***F_z_ = F_z_(I)***   1. Compute the location of edges (E) and their weights (**E_w_):**   $\boldsymbol{E}\left( \boldsymbol{i} \right)\boldsymbol{=}\frac{\boldsymbol{Z}\left( \boldsymbol{i} \right)\boldsymbol{+Z(i+1)}}{\boldsymbol{2}}$  $\boldsymbol{E}_{\boldsymbol{w}}\left( \boldsymbol{i} \right)\boldsymbol{= abs(}\boldsymbol{F}_{\boldsymbol{z}}\left( \boldsymbol{i} \right)\boldsymbol{-}\boldsymbol{F}_{\boldsymbol{z}}\left( \boldsymbol{i+1} \right)\boldsymbol{)}$**, where i is the edge number**   1. Filter edges to define segments boundaries based on their weights   **E = E(top(E_w_))** |

**Figure S13**

**Effect of the Savitzky-Golay filter on the CNAtra performance.** CNAtra is applied to simulated CNA datasets (#1 to #4) with (SG) or without (No-SG) smoothing the RD signal using Savitzky-Golay filter. The bar plot showed the number of true-positive (green) and false positive (purple) FAs in different experiments using simulated datasets.


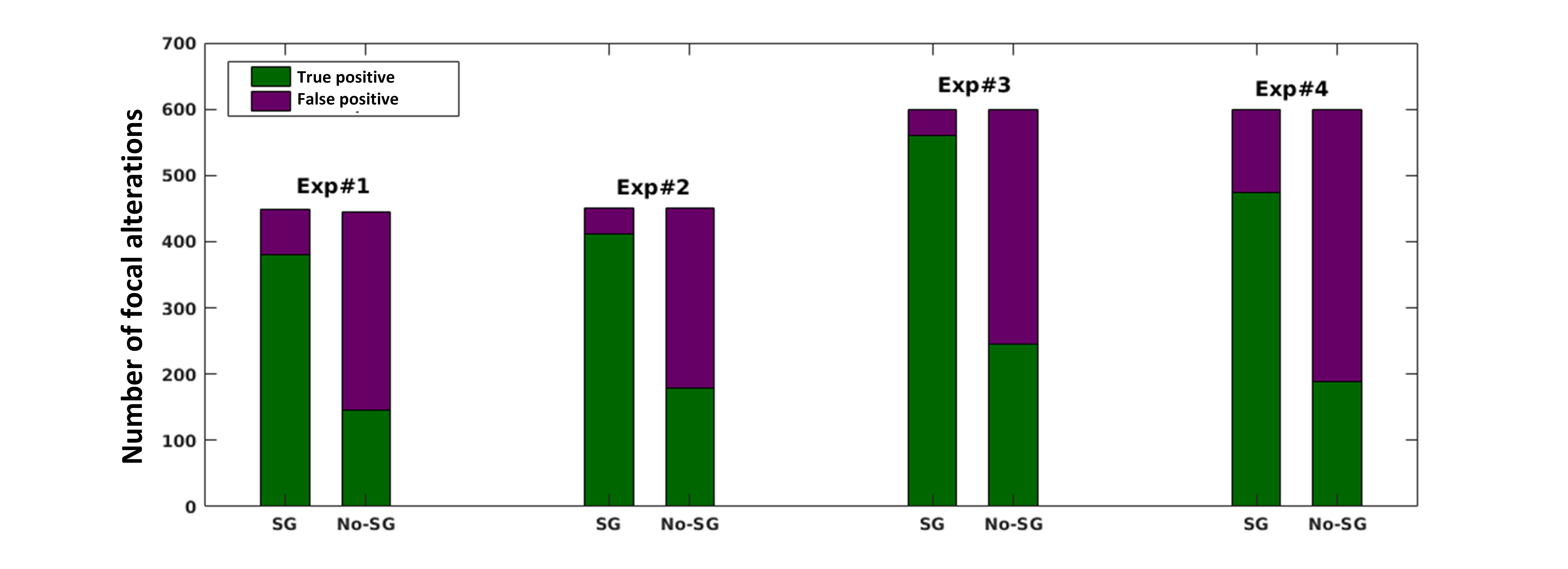


**Figure S14**

**Pseudocode for IB assembly.**

| **IB Assembly Algorithm** |
| --- |
| **Input:**  **F**: RD signal of a chromosome  **[S_s_, S_e_]**: starting and ending bins of each segment of a segment set $\boldsymbol{(S)}$  **Parameters:**  **d_min_:** user-defined minimum IB width (default = 1Mb)  **Output:**  [**FS_s_, FS_e_]**: starting and ending bins of each long segment   1. *Compute the attributes of each segment such as the CN and the state/interval (I) of each segment* $(j)$*:*   ***RD(j) = median(F[S_s_(j), S_e_(j)])***  ***W(j) = S_e_(j) – S_s_(j)+1***  $\boldsymbol{CN}\left( \boldsymbol{j} \right)\boldsymbol{=RD}\left( \boldsymbol{j} \right)\boldsymbol{\times}\frac{\boldsymbol{2}}{\boldsymbol{CNR}}$  $\boldsymbol{I}\left( \boldsymbol{j} \right)\boldsymbol{=RoundNearestInteger(CN}\left( \boldsymbol{j} \right)\boldsymbol{)}$   1. *Filter out homogenous deletion* $\left( \boldsymbol{I=0} \right)$***.*** 2. *Merge the adjacent segments of same copy number state to build the initial segments* ***S1 (top panel Supplementary Fig. S14)****.* 3. *Merge the initial segments (****S1****) iteratively to maximize the intra-segment similarity and build the segments* ***FS (bottom panel Supplementary Fig. S14)****:*   *(i) Merging the small segments to one of its long surroundings:*  ­  a) Divide segments into two groups: long segments $\boldsymbol{(LS)}$ with $(\boldsymbol{W \geq}\boldsymbol{d}_{\boldsymbol{min}}$**)** and short segments **(*SS*)** with $(\boldsymbol{W <}\boldsymbol{d}_{\boldsymbol{min}}\boldsymbol{)}$***.***  b) Assign a merging label $\boldsymbol{(L)}$, starting from 1, to long segments: consecutive long  segments with the same CN interval number (***I***) have the same label.  ***- For j in LS***  ***If (I(j) == I(j+1))***  ***L(j+1) = L(j)***  ***Else***  ***L(j+1) = L(j) + 1***  ***End***  ***End***  c) Short segments between consecutive long segments are merged into one of their surrounding segments or grouped to make a new segment based on their copy numbers.  ***- While (#unlabeled SS != 0)***  ***- Find a group of unlabeled segments (U) between two labeled LS (x, y).***  ***- Assign the group*** ***to a label of their neighbor long segment L(x) or L(y) or to***  ***a new label*** $\boldsymbol{L}_{\boldsymbol{n}}$***.***  ***- CN(u) = majority (CN(i)) where i ∈ U***  ***- If( CN(x) == CN(y))***  ***If( CN(x) == CN(u))***  ***L(u) = L(y) = L(x)***  ***Else***  ***L(u) =*** $\boldsymbol{L}_{\boldsymbol{n}}$  ***End***  ***Else***  ***If(CN(u) == CN(x))***  ***L(u) = L(x)***  ***Else if (CN(u) == CN(y))***  ***L(u) = L(y)***  ***Else***  ***L(u) =*** $\boldsymbol{L}_{\boldsymbol{n}}$  ***End***  ***End***  d) Merge segments having the same label to form the new segments (*S2*).    *(ii) Merge relatively short segment if it falls between two long segments with similar CN:*  ***- For j in S2***  ***Find previous segment (x) and next segment (y)***  ***If (I(x) == I(y)) & (*** $\frac{\boldsymbol{W(j)}}{\boldsymbol{W}\left( \boldsymbol{x} \right)\boldsymbol{+W(y)}}$ ***< 0.05)***  ***L(j) = L(y) = L(x)***  ***End***  ***- Merge segments with the same label to construct the final segments***  *[****FS_s_, FS_e_] (bottom panel Supplementary Fig. S14).*** |

**Figure S15**

**Visual representation of the IB assembly algorithm for low-coverage cancer datasets.** The top panel shows initial segments after simple merging of the adjacent segments with the same CN state for A427 **(a)**, CHP-212 **(b)**, HepG2 **(c)** and NCI-H82 **(d)**. The bottom panel illustrates the final IBs after applying the IB assembly algorithm.


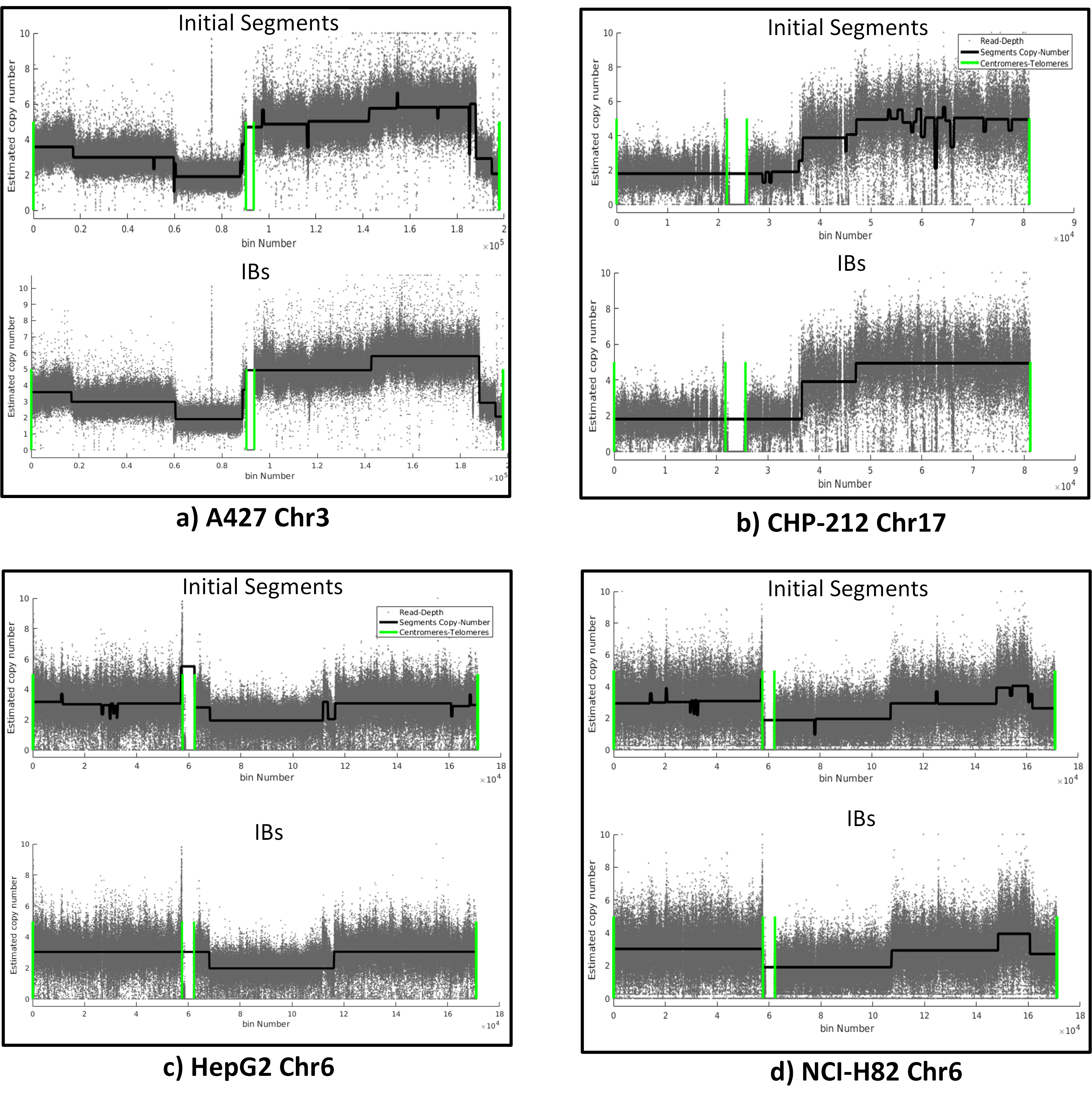


**Figure S16**

**Effect of bin size of RD signal on the false discovery rate (FDR) using HG00119 diploid normal WGS data**. Larger bin size results in low FDR.

**
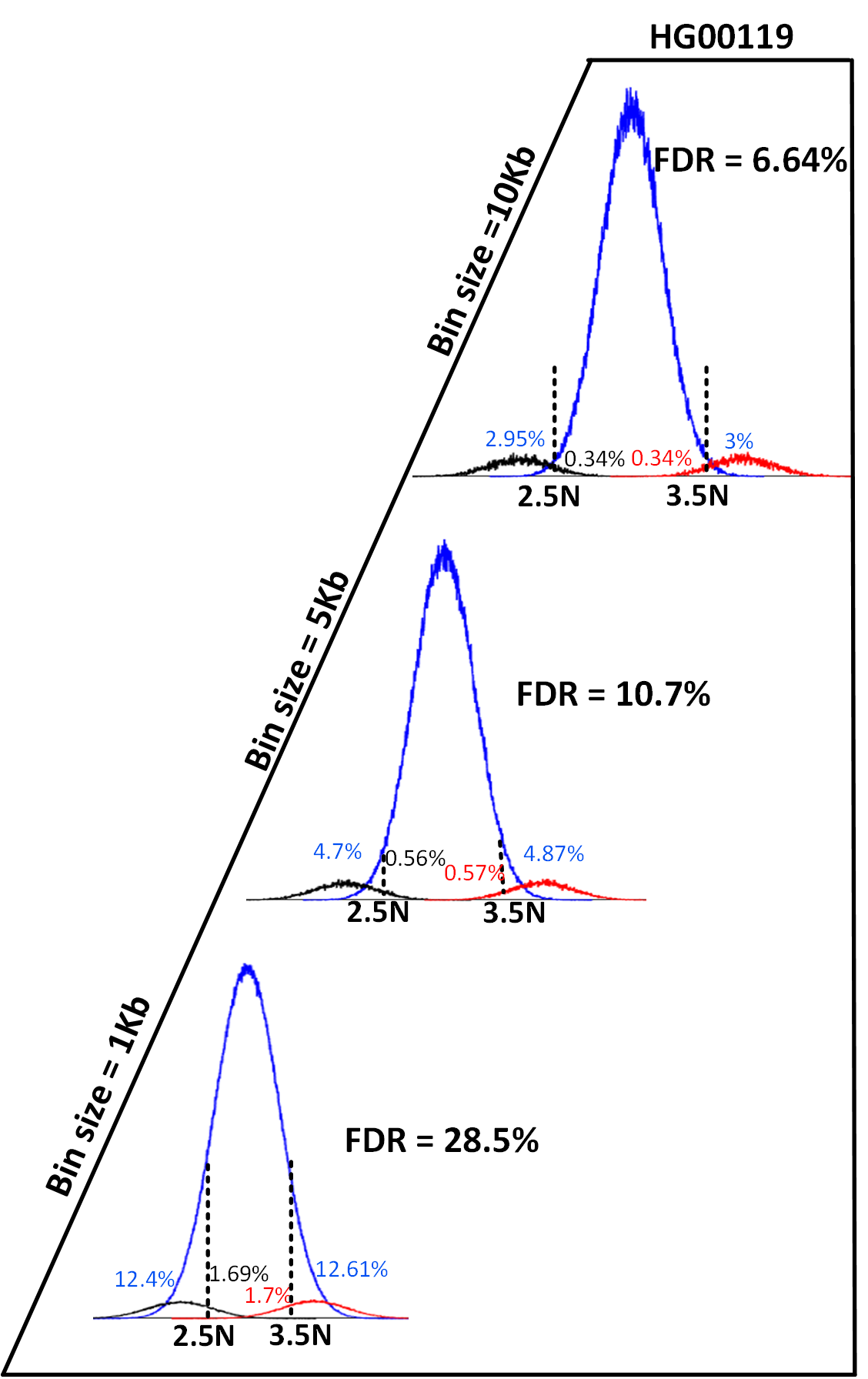
**

**Figure S17**

**Modeling the relationship between the data coverage versus deletion threshold (left) and amplification threshold (right).** Both exponential regression model ‘exp2’ (black line) and power decay model ‘power2’ (blue line) can be utilized for estimating the coverage-dependent CNAtra parameters.


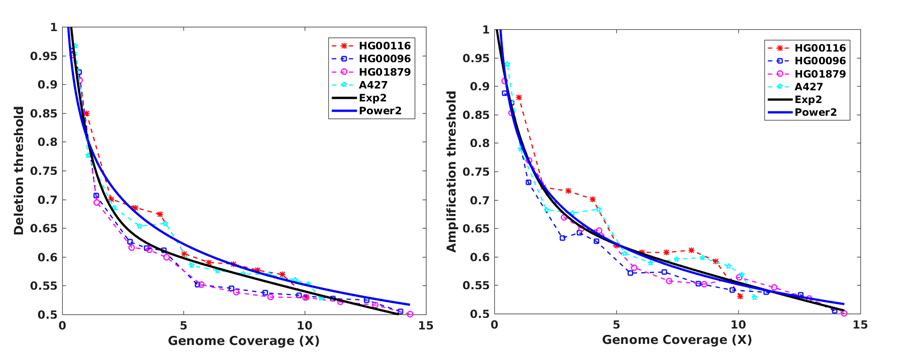


**Figure S18**

**CNVnator results vary significantly with different bin-size options.** CNVnator results of Chr 17 at different bin-size in IMR-32 are plotted. Red, black and gray dots are bins belonging to focally-amplified, focally-deleted and CN-neutral regions. The blue line represents the copy number of each CNA where any amplitude transition indicates a new CNA region. At 1-kb binning, CNVnator can detect FA1, but it also results in oversegmentation of LCV (detected as IB2 by CNAtra) into many CNAs with similar copy number due to short-term variations. At 10-kb binning, the effects of wave artifacts have been lessened resulting in the detection of larger CNAs by CNVnator. However, 10-kb binning results in missing some focal amplifications, such as FA1, which has been detected at 1-kb binning.


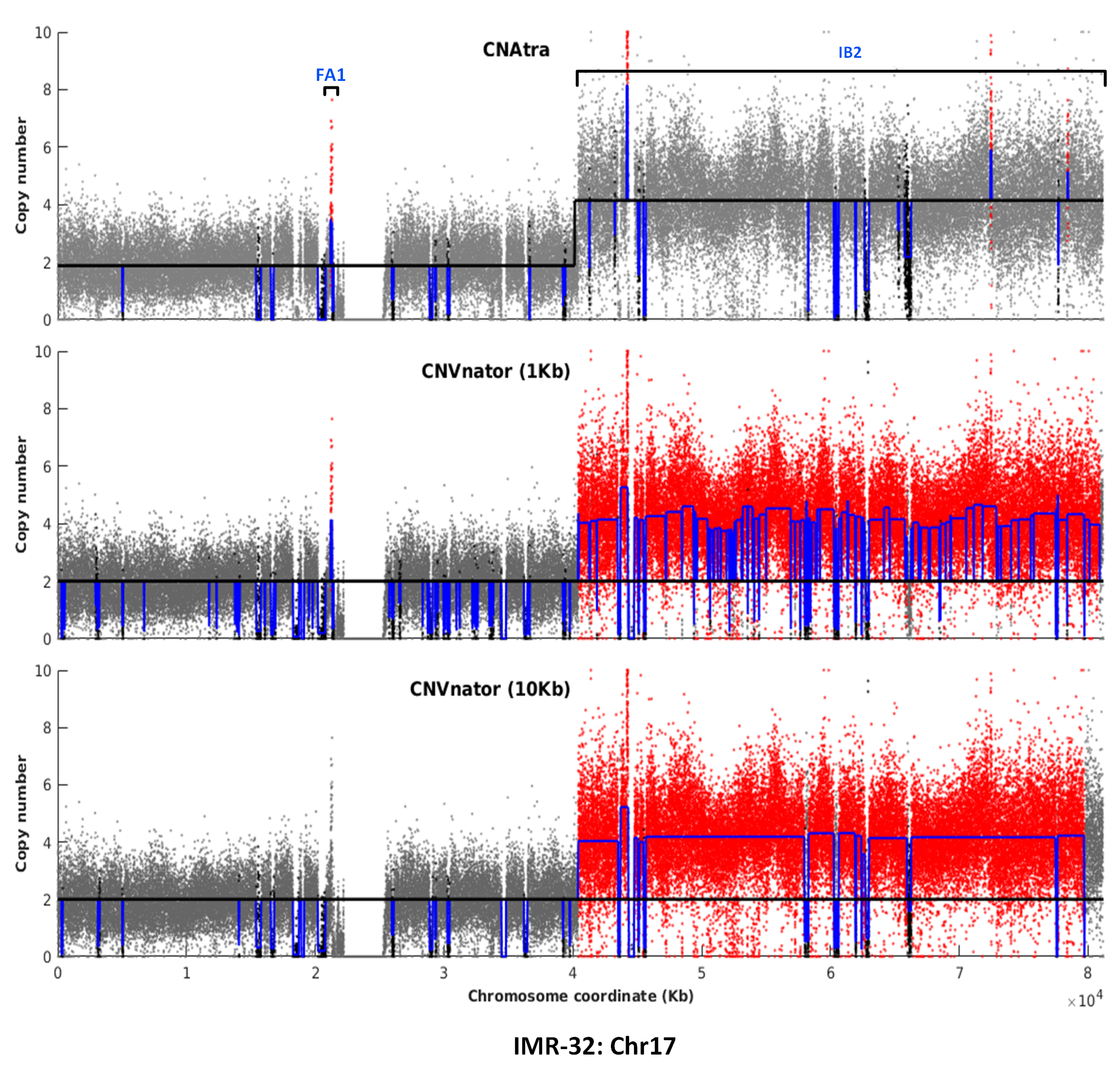


**Figure S19**

**Effect of different ploidy assumption on the CNA calling by FREEC illustrated using Chr 12 of NCI-H82.** FREEC output is dependent on user-defined ploidy level of the cell line. Different ploidy assumptions result in different segments of the RD signal. For example, some large segments under diploid assumption are divided into smaller segments under higher ploidy (triploid/tetraploid) assumptions. This results in false assessment of these segments. Red, black and gray dots are bins belonging to focally-amplified, focally-deleted and CN-neutral regions. The blue line represents the copy number of each CNA where any amplitude transition indicates a new CNA region.


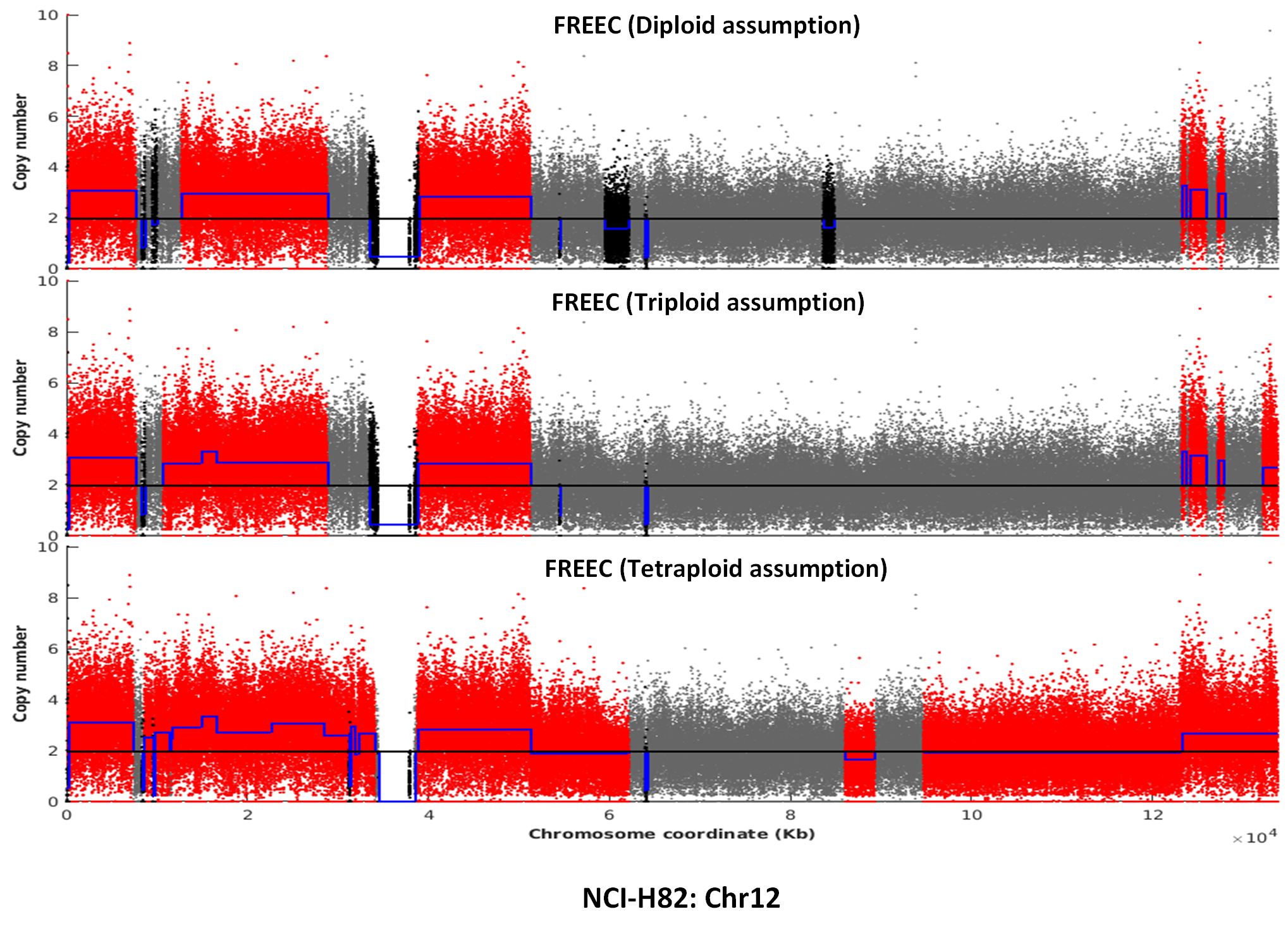


**References**

1. Benjamini, Y. and T.P. Speed, *Summarizing and correcting the GC content bias in high-throughput sequencing.* Nucleic Acids Res, 2012. **40**(10): p. e72.

2. Karimzadeh, M., et al., *Umap and Bismap: quantifying genome and methylome mappability.* Nucleic Acids Res, 2018.

3. Abyzov, A., et al., *CNVnator: an approach to discover, genotype, and characterize typical and atypical CNVs from family and population genome sequencing.* Genome Res, 2011. **21**(6): p. 974-84.

4. Miller, C.A., et al., *ReadDepth: a parallel R package for detecting copy number alterations from short sequencing reads.* PLoS One, 2011. **6**(1): p. e16327.

5. Steinier, J., Y. Termonia, and J. Deltour, *Smoothing and differentiation of data by simplified least square procedure.* Anal Chem, 1972. **44**(11): p. 1906-9.

6. Azami, H., K. Mohammadi, and B. Bozorgtabar, *An improved signal segmentation using moving average and Savitzky-Golay filter.* Journal of Signal and Information Processing, 2012. **3**(1).

7. Kundaje, A., *A comprehensive collection of signal artifact blacklist regions in the human genome.* ENCODE.[hg19-blacklist-README. doc-EBI]. Available online at, 2013.

8. Magi, A., T. Pippucci, and C. Sidore, *XCAVATOR: accurate detection and genotyping of copy number variants from second and third generation whole-genome sequencing experiments.* BMC Genomics, 2017. **18**(1): p. 747.

9. Wang, X., H. Chen, and N.R. Zhang, *DNA copy number profiling using single-cell sequencing.* Brief Bioinform, 2018. **19**(5): p. 731-736.
